# Decoding the genetic drivers of marine bacterial blooms through comparative genomics

**DOI:** 10.1186/s40168-025-02182-y

**Published:** 2025-10-01

**Authors:** Xavier Rey-Velasco, Adrià Auladell, Ona Deulofeu-Capo, Daniel Lundin, Jarone Pinhassi, Isabel Ferrera, Olga Sánchez, Josep M. Gasol

**Affiliations:** 1 Department of Marine Biology and Oceanography, Institut de Ciències del Mar (CSIC), Barcelona, Catalunya 08003 Spain; 2 Institute of Evolutionary Biology (CSIC-UPF), Barcelona, Catalunya 08003 Spain; 3 Centre for Ecology and Evolution in Microbial Model Systems (EEMiS), Linnaeus University, Kalmar, Sweden; 4 Centro Oceanográfico de Málaga, IEO-CSIC, Málaga, Spain; 5 Departament of Genetics and Microbiology, Facultat de Biociències, Universitat Autònoma de Barcelona, Bellaterra, 08193 Spain

**Keywords:** Bloom, Bacteria, Comparative genomics, Functional genes

## Abstract

**Background:**

While oligotrophic bacteria are known to dominate most marine microbial habitats, under certain conditions, such as during phytoplankton blooms, copiotrophs can dramatically increase in abundance and reach towering proportions of the bacterial communities. We are uncertain whether the bacteria exhibiting this capacity, which we denote as “bloomers,” have specific functional characteristics or if, instead, they are randomly selected from the broader pool of copiotrophs. To explore the genomic determinants of this ecological trait, we conducted a comparative genomic analysis of bacterial genomes from microcosm experiments where grazer and viral presence was reduced and nutrient availability was increased, conditions that triggered bacterial blooms.

**Results:**

We tested which functional genes were overrepresented in the bacteria that responded to the treatments, examining a total of 305 genomes from isolates and metagenome-assembled genomes (MAGs) that were categorized as copiotrophs or oligotrophs according to their codon usage bias (CUB). The responsive bacteria were enriched in genes related to transcriptional regulation in response to stimuli (mostly via two-component systems), transport, secretion, cell protection, catabolism of sugars and amino acids, and membrane/cell wall biosynthesis. These genes confer on them capabilities for adhesion, biofilm formation, resistance to stress, quorum sensing, chemotaxis, nutrient uptake, and fast replication. They were overrepresented mainly in copiotrophic genomes from the families *Alteromonadaceae*, *Vibrionaceae*, *Rhodobacteraceae*, *Sphingomonadaceae*, and *Flavobacteriaceae*. Additionally, we found that these responsive bacteria, when abundant, could affect biogeochemical cycling, particularly the phosphorus cycle.

**Conclusions:**

In this study, we provide insights into the functional characteristics that enable certain bacteria to rapidly respond to changes in the environment and bloom. We also hint at the ecological meaning and implications of these phenomena that could affect biogeochemical cycles in the oceans.

Video Abstract

**Supplementary information:**

The online version contains supplementary material available at 10.1186/s40168-025-02182-y.

## Introduction

Microbes dominate the ocean [1, 2], exhibiting a vast metabolic diversity and driving key biogeochemical cycles [3]. Among them, heterotrophic bacteria play a crucial ecological role by supplying organic matter to higher trophic levels through the microbial food web [4, 5]. Marine bacteria have been classified according to their trophic strategy as either oligotrophs, which thrive in nutrient-poor environments, or copiotrophs, which develop in nutrient-rich conditions [6, 7]. Rather than two separate categories, copiotrophy and oligotrophy are two endpoints of a continuum: a given bacterium can be in between or in different parts of the gradient depending on a set of given environmental conditions [8–10]. Under most conditions, marine plankton communities are dominated by oligotrophs, such as members of the SAR11 clade, that develop better in nutrient-poor conditions [11], while copiotrophs are often part of the “rare biosphere” [12]. Despite being rare, copiotrophic bacteria are more important than what their numbers suggest in terms of carbon turnover [13] and carry out energetically expensive functions that are essential for the maintenance of the total community (e.g., making iron available through siderophores or nitrogen fixation) [14].

Overall, copiotrophs and oligotrophs have differential genomic characteristics. The genomes of copiotrophs tend to be larger [15], contain many copies of the ribosomal operon [16], and have higher G + C content compared to those of oligotrophs [8]. Also, they have a high codon usage bias (CUB), i.e., they use specific codons in genes coding for ribosomal proteins that enhance their translation rate, thereby supporting their elevated potential growth rates [17, 18]. Oligotrophic bacterial genomes are more streamlined [8]; they have evolved to minimize size and complexity while retaining essential functions.

Functionally, copiotrophic genomes are enriched in genes related to transcriptional regulation, signal transduction (i.e., reaction to environmental stimuli), defense from antimicrobials or oxidants, motility, low-affinity transport, and metabolism of carbohydrates, amino acids, and inorganic ions. While oligotrophs tend to retain genes related to energy processes, repair, post-translational modifications, secondary metabolites, high-affinity transport, and synthesis of carbohydrates, lipids, proteins, and RNA [7, 17, 19–22]. Copiotrophic bacteria tend to remain at low abundances partly because their larger cell size and high growth rates make them more susceptible to predators [19, 23–25] and their enrichment in membrane proteins makes them good targets for viruses [19, 23, 24, 26, 27].

The scarcity of nutrients (mostly C, N, and P) in the sea is likely the most relevant factor limiting marine bacterial growth, while light can also influence the growth rates of some taxa [28–31]. In fact, it has repeatedly been observed that certain copiotrophs quickly react to sudden events when resource availability is increased, such as during phytoplankton blooms. In these processes, they might reach very high growth rates and, at least for short times, rise from the “rare biosphere” to become substantial components of the community [23, 32–36]. The rapidly growing bacteria that participate in these bacterial blooms—which we here denote “bloomers”—can alter the structure of the bacterial community and drive biogeochemical cycles while they are abundant, highlighting the ecological relevance of these phenomena [37]. Despite the importance of these events, there are no specific studies that have identified the genomic functions involved in promoting the bacterial blooms or explored their potential impact on biogeochemical cycling. We additionally do not know whether copiotrophs bloom stochastically (i.e., any given copiotroph can bloom when, e.g., high amounts of nutrients are available) or if certain taxa are better prepared to do so. We hypothesize that not only nutrient availability but also viral lysis, protist predation, and light are factors that influence the occurrence of bacterial blooms. Additionally, we propose that those taxa more enriched in functions typically associated with copiotrophy are better prepared to bloom than the rest.

Here, we present a comparative genomic analysis of isolate genomes and metagenome-assembled genomes (MAGs) obtained from manipulation experiments that induced bacterial blooms by altering the impact of grazers, viruses, light, and resource availability on the bacterial community dynamics of the Blanes Bay Microbial Observatory (BBMO, NW Mediterranean) over a seasonal cycle. We examined bacterial growth responses to the manipulations and their relationship with functional gene categories in order to (i) identify which genomic populations exhibited high growth rates; (ii) examine the functions associated with rapid responses to sudden changes, compare them between treatments, and assess their distribution across the community; (iii) identify bacteria that reached abundant proportions of the population after fast growth (i.e., those that bloomed) and determine whether they possess differential genomic characteristics compared to the rest of copiotrophs; and (iv) determine whether the responsive bacteria carry genes potentially affecting major biogeochemical cycles in the ocean. In this study, we use the term “responsive bacteria” to refer to those bacteria that reacted to the treatments with fast growth and increased their relative abundance.

## Materials and methods

**Fig. 1:**
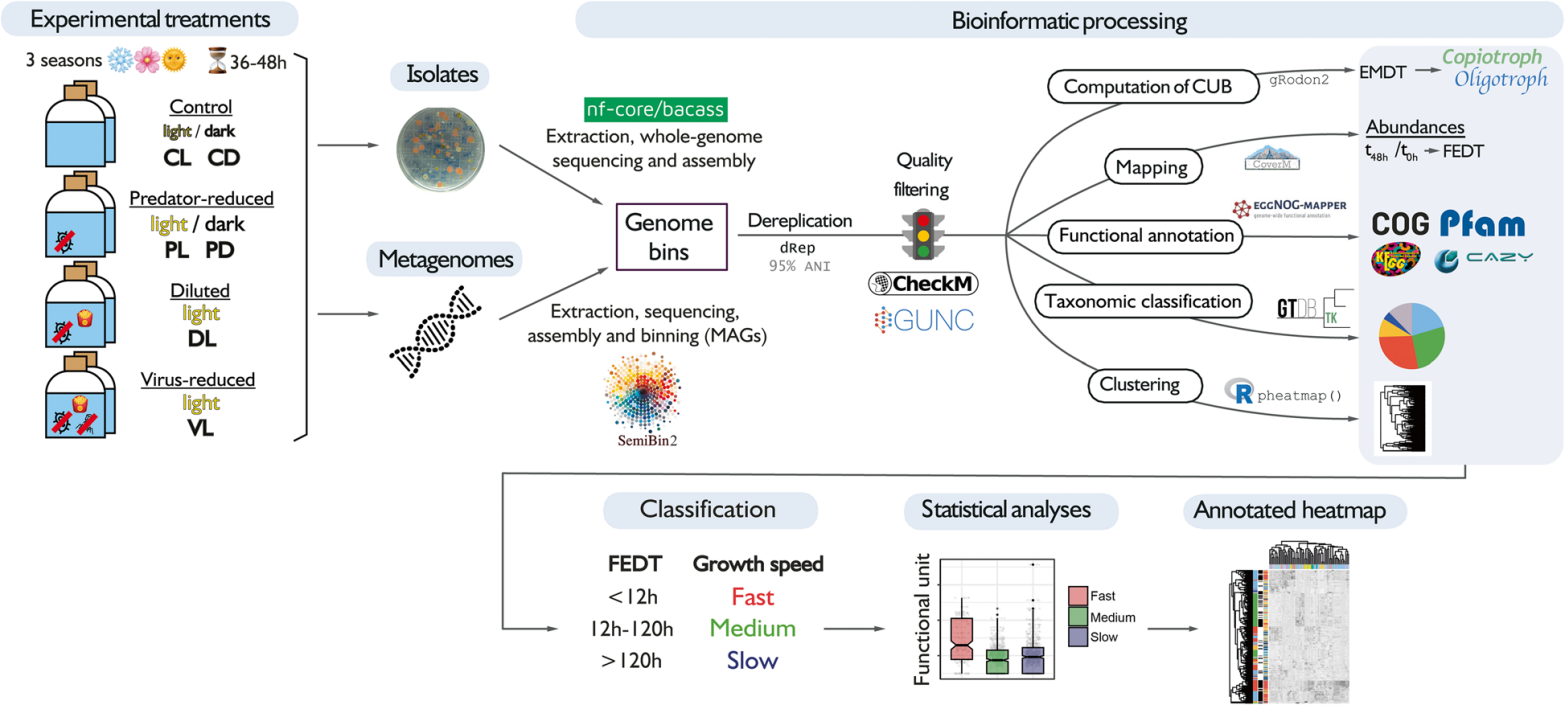
Schematic representation of the methods used in this work. CL = control light, CD = control dark, PL = predator-reduced light, PD = predator-reduced dark, DL = diluted light, VL = virus-reduced light, MAG = metagenome-assembled genome, ANI = average nucleotide identity, CUB = codon usage bias, EMDT = estimated minimum doubling time, FEDT = fold-change–based experimental doubling time

A schematic representation of the methods used in this work can be found in Fig. 1.

### Origin of samples

Surface seawater was collected from the BBMO in the NW Mediterranean (41º40'N, 2º48'E), about 70 km north of Barcelona, and approximately 1 km offshore. The water was collected on the four astronomical seasons: winter (21 February 2017), spring (26 April 2017), summer (5 July 2017), and fall (7 November 2017), filtered in situ through a 200-μm mesh and transported to the laboratory within 2 h.

### Manipulation experiments

Six experimental treatments in each season were set up as described in Sánchez et al. [31]. Briefly, the treatments consisted on (i–ii) unfiltered seawater in light/dark cycles (CL, Control Light) and in the dark (CD, control dark), (iii–iv) seawater prefiltered through a 1-μm filter to remove large predators while preserving most bacteria in light/dark cycles (PL, predator-reduced light) and in the dark (PD, predator-reduced dark), (v) unfiltered seawater diluted 1/4 with 0.2-μm-filtered seawater to reduce predators and increase nutrient availability in light/dark cycles (DL, diluted light), and (vi) unfiltered seawater diluted 1/4 with 30-kDa-filtered seawater to reduce predators and viruses and increase nutrient availability, in light/dark cycles (VL, virus-reduced light). These treatments form a manipulation gradient: the CL and CD experiments are controls, the PL and PD experiments incorporate a reduction in predator presence, and the DL treatment represents an increase in nutrient availability, also adding a reduction in predators. Finally, the VL treatment is the strongest manipulation, adding virus reduction to all the previous effects. The different treatments were incubated in triplicate 9 L Nalgene bottles for up to 48 h at in situ temperature (reported in Table 1 in Sánchez et al. [31]) in a water bath with circulating seawater. Light treatments were limited to photosynthetically active radiation by using two layers of an Ultraphan URUV Farblos Filter and a net, and bottles in dark treatments were covered with several layers of dark plastic.

Samples from each replicate Nalgene bottle were taken for community DNA, bacterial isolation, flow cytometry, inorganic nutrient concentration, and other ancillary data (reported in Ref. [31]) at times 0 h, 12 h, 24 h, and 36 h in summer and winter or 48 h in the fall and spring experiments. For isolation, 1-mL seawater subsamples were mixed with 75-μL dimethyl sulfoxide (DMSO) in cryovials that were stored at − 80 °C in triplicate. Flow-cytometry measurements were done with a FACSCalibur (BectonDickinson) flow cytometer, and discrimination of populations with high nucleic acid content (%HNA) was done as described previously [38].

### Community DNA extraction and sequencing

Samples were prefiltered through a 20-μm mesh to remove large particles, and microbial biomass was concentrated onto 0.2-μm polycarbonate filters using a peristaltic pump. About 2–4 L were filtered from each replicate in all treatments. We extracted the DNA from the filters as described in Massana et al. [39], and this DNA was purified and concentrated using Amicon 100 columns (Millipore), subsequently ethanol-precipitated, and quantified in a NanoDrop-1000 spectrophotometer (Thermo Scientific). DNA was stored at − 80 °C.

For 16S rRNA gene amplicon sequencing, an aliquot of each replicate was PCR-amplified with primers 515F-Y (5′-GTG YCAG CMG CCG CGG TAA) and 926R (5′-CCG YCA ATT YMT TTR AGT TT) from Parada et al. [40] comprising the V4–V5 regions of the 16S rRNA gene. Illumina sequencing was performed using a MiSeq sequencer (2 × 250 bp, Illumina). A first run was sent to the Integrated Microbiome Resource (Halifax, NS, Canada; https://imr.bio), and a second run with problematic amplification samples was sent to the Research and Testing Laboratory (Lubbock, TX, USA; http://rtlgenomics.com/) in order to improve the amplification quality of a set of samples. ASVs were obtained from metabarcoding data using Dada2 [41], and their growth rates were calculated by fitting linear regressions to in-transformed pseudoabundances (relative abundances multiplied by flow cytometry total abundance values) over time, selecting the steepest and most significant slope (*P* < 0.05) to represent maximal exponential growth rates under each condition as described in Deulofeu-Capo et al. [42].

For metagenome sequencing, an aliquot from each sample was processed in a Novaseq 6000 machine at the Centre Nacional d'Anàlisi Genòmica (CNAG) with paired-end fragments of 150 bp. A total of 66 samples were sequenced with an average of 115 million reads (min = 67 M, max = 238 M) each. We obtained metagenomic data for winter and summer (two replicates of the final times) as well as for the spring experiments (three replicates of the final times). We used *illumina-utils* [43] for quality filtering the short reads from the metagenomes with the *iu-filter-quality-minoche* function (default parameters), which removes noisy reads following the method described in Minoche et al. [44].

### Generation of metagenome-assembled genomes

To delineate MAGs, we first assembled the 66 quality-filtered metagenomic samples separately using the metaSPAdes pipeline from SPAdes v.3.15.4 [45]. Three samples could not be run with metaSPAdes due to high memory requirements, so they were assembled with MEGAHIT v.1.2.8 [46]. We then used SemiBin2 v.1.5.1 [47] to perform a multi-sample binning. With this procedure, all samples were binned individually using co-abundance information from a representative subset of the samples. For this, we first created a concatenated fasta file containing all assembled contigs longer than 1000 bp and then mapped it to a selection of 18 samples including all the conditions in the experiments (there were samples from all treatments, times, and seasons). After mapping with Bowtie2 v.2.5.1 [48], we used SAMtools v.1.16.1 [49] to convert the resulting sam files to sorted bams and index them. Finally, we used these data to perform multi-sample binning with the SemiBin *multi_easy_bin* command, obtaining 18,005 bins.

### Culturing, selection, extraction, and sequencing of isolates

We obtained a collection of 1643 isolates by culturing 100 µL of seawater in Marine Agar and Marine Reasoner’s 2A Agar from the initial and final times of the different experiments, and their near-complete 16S rRNA gene was amplified by PCR and sequenced using Sanger sequencing as described in Rey-Velasco et al. [50]. In order to select potential bloomers among the isolates, we compared the V4–V5 regions of the 16S rRNA gene sequences of the isolates with the ASV sequences covering the same region using BLASTn v. 2.12.0 + [51]. We selected 31 isolate strains as potential bloomers whose V4–V5 regions of the 16S rRNA gene was 100% similar to ASVs that had changed in relative abundance from < 1% at the initial time of the experiments to > 1% in the final time of the same experiment and had doubling times of at most 1 day.

The genomic DNA of the 31 selected strains was extracted using the DNeasy Blood&Tissue Kit (Qiagen) following the manufacturer’s recommendations, and their integrity and concentration were checked using DNA gel electrophoresis and a Qubit 1 Fluorometer (Invitrogen), respectively. Samples were then sent to the Centre Nacional d'Anàlisi Genòmica (CNAG) for further quality control, library preparation, and whole-genome sequencing with an Illumina MiSeq sequencer (2 × 300 bp, Illumina).

### Generation of isolated genomes and quality control

To assemble the genomes of the isolates, we used the Nextflow pipeline bacass v.2.0.0 [52] from the nf-core framework [53]. The pipeline runs an automated workflow that uses Skewer [54] to quality trim the reads, performs basic sequencing QC using FastQC [55], and assembles the reads with Unicycler [56]. Assembly contamination was checked with Kraken2 [57], and its quality was assessed using QUAST [58]. Additionally, the protein-coding sequence (CDS) of the resulting assembly was annotated with Prokka [59].

### Combination of MAGs with isolated genomes and quality filtering

We pooled together the 18,005 bins obtained from the metagenomes with the isolate genomes and dereplicated them using dRep v.3.4.0 [60] with a 95% ANI threshold. Then, they were quality-filtered with CheckM v.1.2.1 [61] using 75% minimum completeness and 5% maximum contamination. These thresholds were chosen to ensure that the genomes would have enough quality to allow for a significant functional comparison. After these steps, we obtained 335 good-quality genomes, including MAGs and isolate genomes. These genomes were then analyzed with GUNC v.1.0.5 [62] to unveil possible chimeras, and we detected 31 of them (clade separation score > 0.45). We then removed, from each genome, the contigs where GUNC reported that all genes affiliated with species different than the predominant. After this curation step, 26 genomes out of the 31 passed GUNC detection of chimerism and CheckM quality filtering with the same thresholds as before. Thus, the final dataset of good-quality, nonchimeric, dereplicated genomes contained 330 genomes.

### Taxonomic and functional annotation of the genomes

All genomes’ CDSs were obtained with Prokka v.1.14.6 [59] and then functionally annotated using eggNOG-mapper v.2.1.9 [63] based on eggNOG orthology data [64] and DIAMOND sequence searches [65]. The databases used for this annotation were Clusters of Orthologous Groups (COGs) [66], the Pfam database [67], the Kyoto Encyclopedia of Genes and Genomes (KEGG) [68], and the Carbohydrate Active Enzyme (CAZY) database [69]. For taxonomic classification, GTDB-Tk v.2.3.2 [70–76] was used relying on release 214 of the GTDB database [77]. All these pipelines and CheckM were run using custom makefiles available on GitHub (https://github.com/erikrikarddaniel/biomakefiles).

Once we obtained the taxonomic classification, and because this study is based on the functional dynamics of heterotrophic bacteria, genomes from archaea and cyanobacteria were excluded, which resulted in a total of 305 genomes left for further analyses.

### Computation of genome abundances and experimental doubling times

We obtained relative abundances of our genomes by mapping them to the metagenomes using CoverM with default parameters (https://github.com/wwood/CoverM). We used a 25% breadth of coverage as a threshold to decide whether a genome was present or not in a given sample.

We calculated the fold-change between the initial and final times in each season and treatment for all genomic populations. Then, we inferred fold-change–based experimental growth rates as the natural logarithm of the fold change divided by the duration of the experiment in hours and fold-change–based experimental doubling times (FEDTs) as the natural logarithm of two divided by the growth rate (e.g., Madigan et al. [78]). We add the phrase “fold-change–based” because our calculations were based on only two time points without the possibility of observing a proper growth curve, and thus, we cannot use the term “experimental doubling time” without any clarification here. In cases where a given genomic population had an abundance value at the initial time but was not detected at the final time, an arbitrary fold-change of 0.1 was assigned, resulting in a negative FEDT, which was transformed, also arbitrarily, into 500 h. Similarly, when the final abundance was > 0 at the final time but 0 at the initial time, an arbitrary fold-change of 100 was assigned.

### Estimation of minimum doubling times and trophic strategy

We computed estimated minimum doubling times (EMDTs) in R v.4.1.3 [79] and RStudio environment [80] using the gRodon package [17] in *partial* mode. The algorithm takes into account CUB statistics calculated from the difference in codon usage of highly expressed genes compared to the rest of the genes in each organism. We classified the organisms into copiotrophs or oligotrophs if they had EMDTs < 5 h or ≥ 5 h, respectively, following Weissmann et al. analysis [17]. In order to test the validity of the EMDTs, we compared the estimated and experimental minimum doubling times (i.e., the EMDTs to the minimum FEDTs) of each genome by calculating the Pearson correlation coefficient between them.

### Statistical analyses

All analyses were carried out in the R software v.4.1.3 [79] and RStudio environment [80]. Packages *tidyverse* v.1.3.2 [81], *magrittr* v.2.0.3 [82], and *qdap* v.2.4.3 [83] were used for data treatment, and most plots were made with *ggplot2* v.3.4.0 [84], sometimes arranging them using *ggfortify* v.0.4.15 [85].

In all statistical analyses, we first checked if the data followed a normal distribution with the Shapiro–Wilk test. Since the data could never be considered in a normal distribution, all comparisons were made with Wilcoxon rank sum tests (when comparing two groups) or Pairwise Wilcoxon rank sum tests (when comparing more than two groups) utilizing the Benjamini–Hochberg correction [86].

The general workflow was as follows: using the output from eggNOG-mapper, we created tables containing, for each genome, the counts of genes affiliating with every COG, COG categories, Pfams, KEGG Orthologs (KOs), CAZYs, and CAZY categories. To account for differences in the quantity of genes in each genome, the count tables were normalized by the number of genes in each genome predicted by CheckM. Multiplying these proportions by 100, we obtained a table displaying the percentage of genes that each functional unit (FU) represented in each genome. Here, we define a FU as a set of genes or proteins that are grouped based on their shared functions, evolutionary relationships, or biochemical pathways; therefore, each COG, Pfam, KO, or CAZY is a FU. COG categories, encompassing wider classifications such as “translation” or “ribosome biogenesis,” are not considered FUs in this work. We used these tables to test for functional differences between copiotrophs and oligotrophs in our dataset.

To determine which functions characterized the responsive bacteria, for each genome and treatment, we chose the FEDT value of the season in which it was minimal. Then, we grouped the bacteria into fast-, medium-, or slow-growers if they had FEDTs of less than 12 h, between 12 and 120 h, or higher than 120 h, respectively. These thresholds were defined according to the overall distribution of FEDTs in our dataset. Using the gene count tables, we tested for functional differences (including COG categories, individual COGs, Pfams, KOs, CAZY categories, and individual CAZYs) between these fast, medium, and slow-growing genomic populations in all treatments. To reduce noise, we excluded those FUs that amounted to less than 5% when summing up the percentages of all genomes. We compared the functions that explained fast growth in each treatment with an upset plot based on the COGs that were significantly enriched in fast-growers compared to slow-growers in each treatment.

The COGs, Pfams, and KOs that were significantly enriched in fast-growers compared to slow-growers were manually checked and categorized. We then selected a subset of them taking into account the differential enrichment between fast and slow-growers (we selected the most different ones), the treatments where a given gene was significant (we tried to consider functions that were enriched in the fast-growers in all treatments but also those enriched only in certain treatments), and their functional category (we included several genes of all main categories). We computed a clustered heatmap to examine the distribution of these selected genes across taxonomic groups using *pheatmap* v.1.0.12 [87].

On the other hand, a selection of some biogeochemically relevant genes was made based on previous studies (e.g., [88–90]), to test their enrichment in fast-growing bacteria. Genomic (G + C content and genome length calculated with CheckM, CUB, and EMDT calculated with gRodon) and functional differences between bloomers and the rest of the copiotrophs were also tested.

We considered that a genomic population bloomed in a given experiment and season if, besides being a fast-grower (i.e., it had an experimental doubling time shorter than 12 h), it became abundant (i.e., increased in relative abundance from < 1% in the initial time to > 1% in the final time).

## Results

### Contextualization of the data

Our dataset consisted of 305 genomes from various species of heterotrophic bacteria (dereplicated at a 95% ANI threshold), of which 13 came from isolates and 292 from metagenomes (i.e., they were MAGs). Taking into account all genomes, the mean completeness and contamination were 91.45% and 1.05%, respectively, and the number of contigs in each genome ranged from 12 to 493 (Additional file 10: Table S1). Most of the genomes are affiliated with the classes *Bacteroidia* (96 genomes), *Alphaproteobacteria* (87 genomes), and *Gammaproteobacteria* (77 genomes), with some representatives of the *Verrucomicrobiae* (14 genomes), *Acidimicrobia* (seven genomes), and 10 other classes. There were representatives of 83 families, including *Flavobacteriaceae*, *Rhodobacteraceae*, *Alteromonadaceae*, *Sphingomonadaceae*, *Halieaceae*, and *Porticoccaceae* (Additional file 10: Table S1). The gRodon algorithm predicted that there were 175 oligotrophic genomes and 130 copiotrophic genomes based on their codon usage bias (CUB) and their estimated minimum doubling times (EMDTs). These minimum doubling times estimated computationally correlated reasonably well with the ones observed experimentally (based on the results from mapping the genomes to the metagenomes and calculating the genome coverages with CoverM) (linear model *p* < 0.001, adjusted *r*^2^ = 0.35, Additional file 1: Figure S1), albeit with a different magnitude. Copiotrophs had significantly larger genomes than oligotrophs (Wilcoxon rank sum *p* < 0.01); however, there was no significant correlation between genome size and fold-change based experimental doubling time (FEDT) (Pearson correlation adjusted *r*^2^ = 0.074). According to the flow cytometry data, during the experiments, there was an increase in abundance of the whole bacterial community, especially in the summer and fall experiments and in the diluted light (DL; increased in nutrient availability and reduced in predators) and virus-reduced light (VL) treatments (Additional file 2: Figure S2).

On average, 71.28% of the total metagenomic reads mapped to the genomes, with higher values retrieved in the final time data than in the initial times (Additional file 3: Figure S3A). The maximum percentage of mapped reads was 85.97% in the summer VL t_f_ treatment, while the minimum value was 54.16% in the winter DL t_0_. Copiotrophic genomes tended to be more abundant in the final time of the experiments, increasing from the predator-reduced light (PL) to the VL treatments as the degree of manipulation increased (Additional file 3: Figure S3B).

**Fig. 2:**
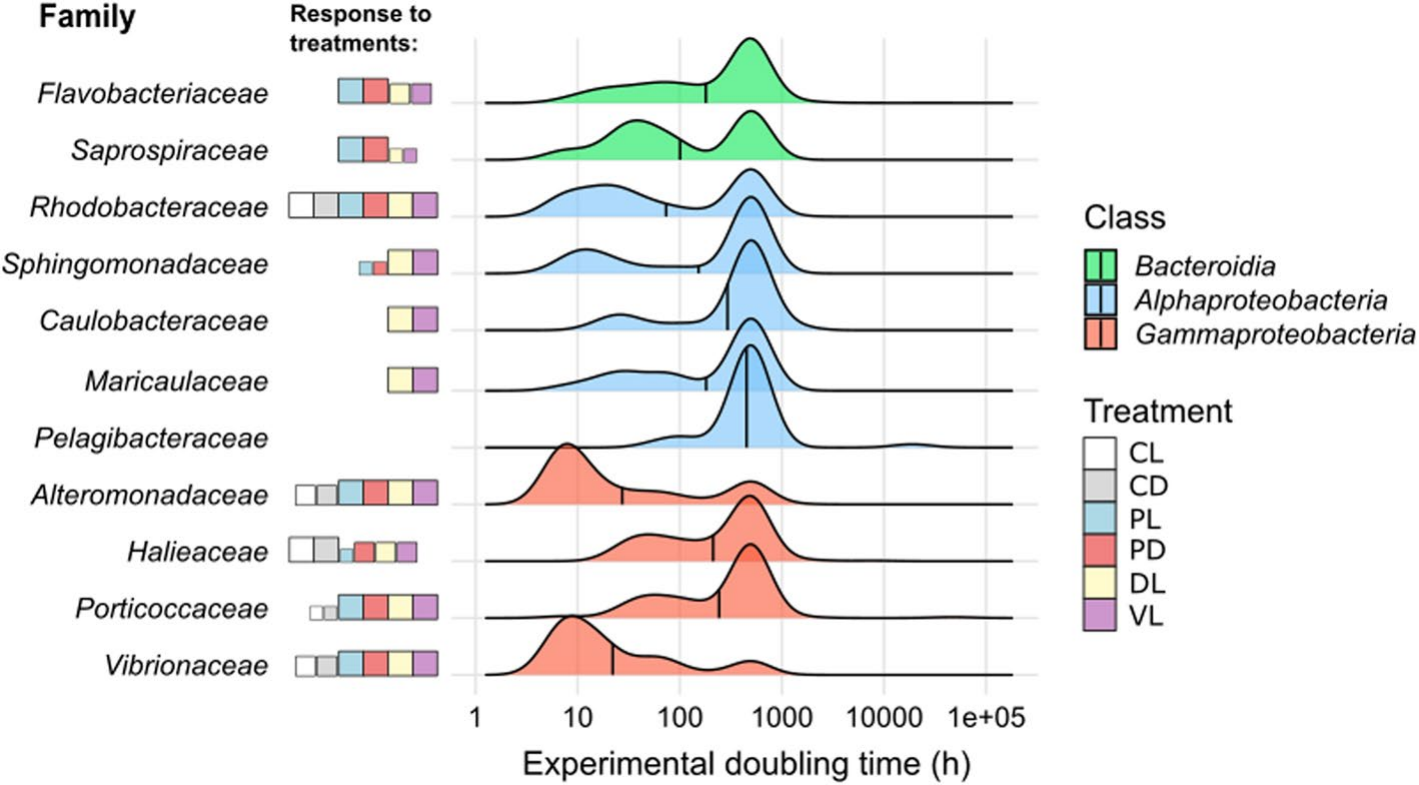
Experimental doubling times of the main families. The *y*-axis shows the different families and, to the left, there are squares indicating which treatments did the specific family have the highest responses (lowest doubling times). The size of the squares is relative to the intensity of the response within each family. The absence of squares indicates that the given family did not respond to any treatment

To examine the bacterial responses to the treatments, we used the abundances inferred from genome coverage and calculated fold-changes between the initial and final times of the experiments. These were converted to fold-change–based experimental doubling times (FEDTs) as explained in the methods. Examining the response of the main families to the treatments, we observed that the genomic populations affiliating with families *Alteromonadaceae* (most frequently, genera *Alteromonas*, *Pseudoalteromonas*, or *Glaciecola*), *Vibrionaceae* (*Vibrio crassostreae*, *V. coralliirubri*, and *Enterovibrio *sp.), and *Rhodobacteraceae* (e.g., *Limimaricola cinnabarinus*, *Nereida ignava*, or *Planktomarina temperata*) reached very low FEDTs (< 12 h) in all the treatments, with higher responses in the predator-reduced, DL and VL treatments (Fig. 2, Additional file 4: Figure S4, Additional file 11: Table S2 contains all data). It is noteworthy that *Alteromonadaceae* and *Vibrionaceae* did not have a unimodal distribution of FEDTs in the control treatments, with a variable response to the different seasons. On the contrary, their response to the manipulation treatments shows a higher peak in the fast region of the doubling times (FEDT ~ 10 h), meaning that almost all populations within these families reacted to the experiments displaying fast growth (Fig. 2). Families *Flavobacteriaceae* (e.g., genera *Polaribacter*, *Patiriisocius*, or *Dokdonia*) and *Saprospiraceae* also displayed considerably low FEDTs, especially in predator-reduced treatments (Fig. 2, Additional file 4: Figure S4). Other families, such as *Sphingomonadaceae*, *Maricaulaceae*, or *Caulobacteraceae* (e.g., *Sphingomonas aquatilis*, *Marinicauda pacifica*, and *Brevundimonas aurantiaca*, respectively), responded almost exclusively to the DL and VL treatments. Although most members of families *Halieaceae* or *Porticoccaceae* did not reach low FEDTs (Fig. 2), some populations showed a slight response to the manipulations (Additional file 4: Figure S4). The *Pelagibacteraceae* family did not respond to the manipulations, although they did show some growth in the control treatments with a minimum of 57 h for a division (Fig. 2, Additional file 4: Figure S4, Additional file 11: Table S2).

### Functions involved in the response to the manipulations

To elucidate which functions were associated with the response to each treatment, we first classified the genomic populations as fast-growing (FEDT < 12 h), medium-growing (12 h < FEDT < 120 h), and slow-growing (FEDT > 120 h; Additional file 10: Table S1) for each experiment. The genomic populations classified as fast-growers were dominated by *Gammaproteobacteria*, *Alphaproteobacteria*, and *Bacteroidia* in all treatments*,* with *Alphaproteobacteria* increasing their abundance as the degree of manipulation became higher. Similarly, the number of genomes classified as fast-growers increased sequentially from the control light (CL; *n* = 19) to the VL treatment (*n* = 45; Additional file 5: Figure S5).

**Fig. 3:**
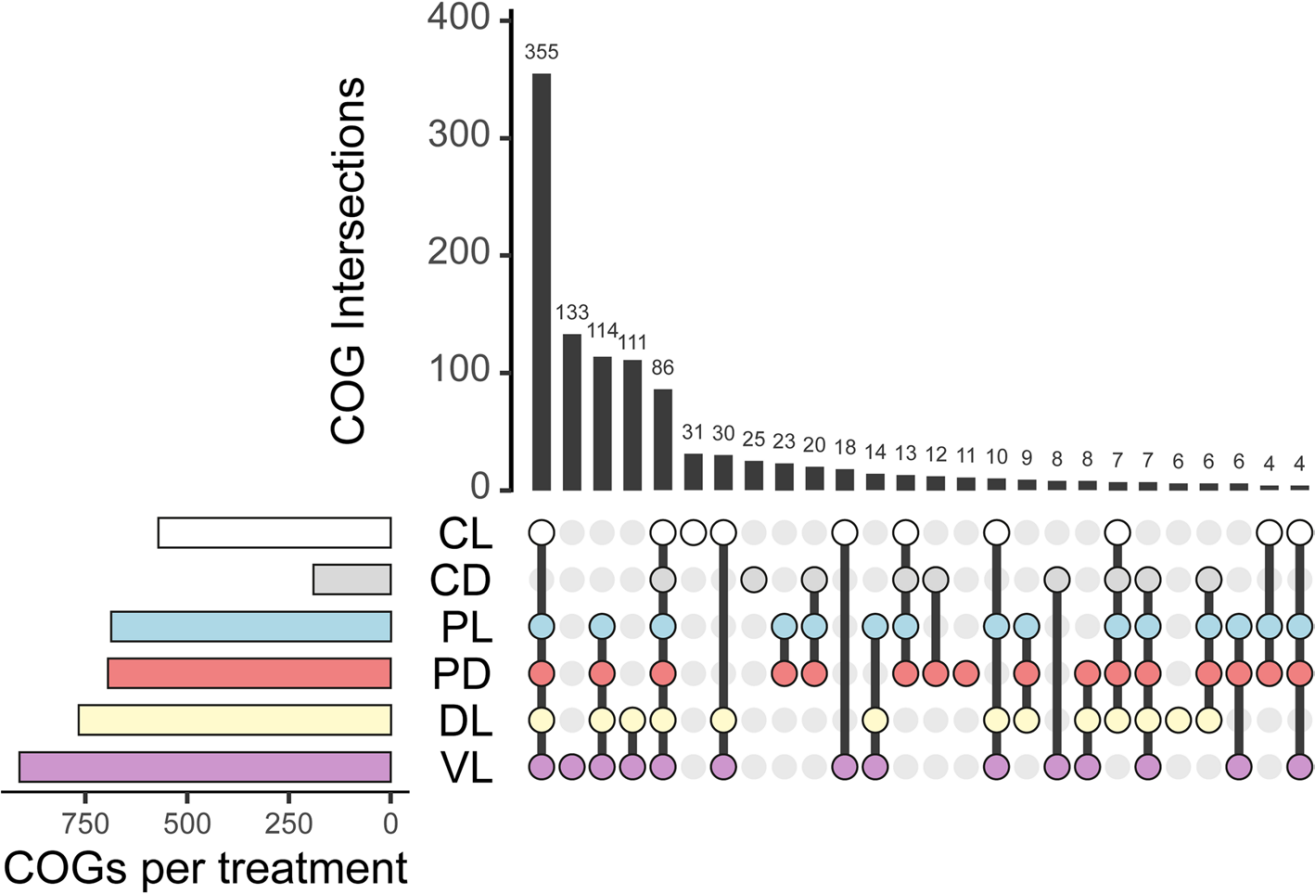
Similarities between the COGs associated with fast growth across treatments. Upset plot representing the COGs that are significantly enriched (Wilcoxon rank sum test, *p* < 0.05) in fast-growers as compared to slow-growers in each treatment. In this plot, the COG intersections indicate the number of COGs that are enriched in the same treatments. The treatments that share these COGs are indicated below. Interactions with less than three COGs have been deleted to simplify the interpretation of the plot. The colored barplot on the left side outlines the total number of COGs that are significantly enriched in each treatment. CL = control light, CD = control dark, PL = predator-reduced light, PD = predator-reduced dark, DL = diluted light, and VL = virus-reduced light

We analyzed the Clusters of Orthologous Genes (COGs) that were overrepresented in fast-growers compared to slow-growers across the different treatments. Most of these fast-growers COGs were shared among treatments CL, PL, PD, DL, and VL, though each experiment had some unique COGs associated with fast growth (the PL experiment had less than three unique COGs; therefore, they are not shown). The VL treatment had the highest number of unique COGs (Wilcoxon rank sum test, *p* < 0.05; Fig. 3). The number of COGs related to the response to the manipulations increased sequentially from CL to VL, with the exception of CD, which had lower numbers of significant COGs than all the other treatments (see side-barplot in Fig. 3) probably because the bacteria that displayed fast growth in this treatment were predicted as oligotrophs (Additional file 10: Table S1).

**Fig. 4:**
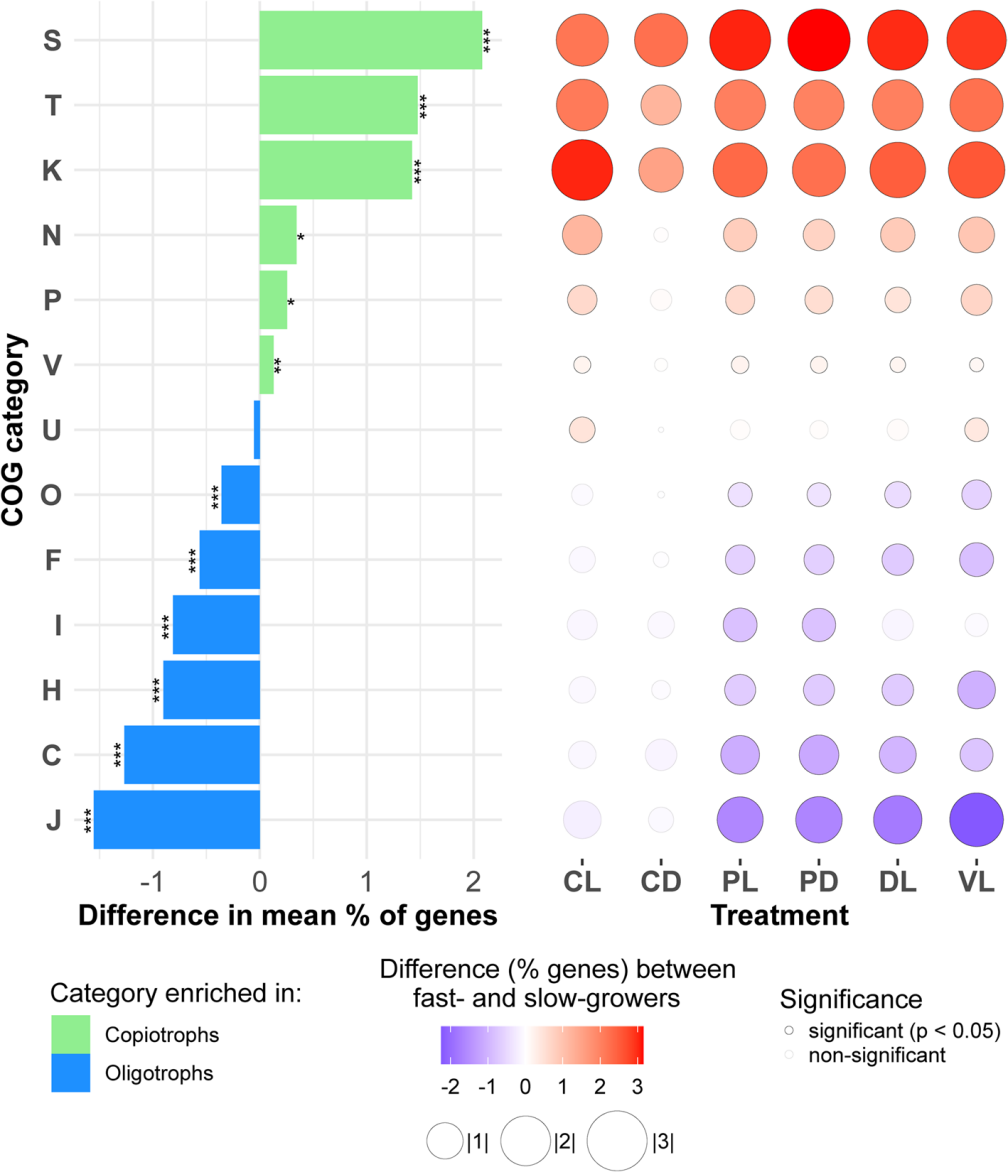
COG categories across treatments. On the left, a barplot shows the enrichment of each COG category in copiotrophs vs. oligotrophs (mean % genes in copiotrophs—mean % of genes in oligotrophs). *p < 0.05, **p < 0.01, and ***p < 0.001. On the right, a bubble plot shows whether each category was significantly enriched in fast-growers versus slow-growers and the intensity of enrichment across treatments. Bubble sizes are set according to the absolute difference between the gene enrichment in fast- and slow-growers. CL = control light, CD = control dark, PL = predator-reduced light, PD = predator-reduced dark, DL = diluted light, VL = virus-reduced light. Codes for COG categories: S = function unknown, T = signal transduction mechanisms, K = transcription, N = cell motility, P = inorganic ion transport and metabolism, V = defense mechanisms, U = intracellular traffic, secretion and vesicular transport; O = post-translational modification, protein turnover, chaperone functions; F = nucleotide transport and metabolism, I = lipid transport and metabolism, H = coenzyme transport and metabolism, C = energy production and conversion, J = Translation, ribosomal structure and biogenesis

We also tested which particular COG categories were overrepresented in fast-growers across treatments and whether these categories were more present in the copiotrophs (genomes with EMDT < 5 h) or in the oligotrophs (genomes with EMDT ≥ 5 h) in our dataset. We observed that in all treatments, fast-growers were enriched in genes of unknown function (S category), genes related to signal transduction mechanisms (T category), and transcription (K category). All these COG categories were also significantly overrepresented in copiotrophs compared to oligotrophs (Wilcoxon rank sum test, *p* < 0.001; Fig. 4). Also, though with less significance and narrower difference, fast-growers had more genes related to cell motility (N category), inorganic ion transport and metabolism (P category), and defense mechanisms (V category) in almost all treatments (Fig. 4).

### Specific functional units involved in fast responses and their distribution across taxonomic groups

To gain a deeper understanding of the mechanisms involved in fast responses to top-down and bottom-up factors, we compared the genomes of fast- and slow-growing bacteria to analyze their differential enrichment in individual functional units (FUs), a term that here comprises Pfams, COGs, and KEGG Orthologs (KOs). From the 2906 FUs that were significantly over- or underrepresented in fast growers in any of the treatments (Additional file 12: Table S3), we selected a set of representative FUs for further study. The selection process involved (1) choosing FUs with the greatest enrichment differences between fast- and slow-grower genomes, (2) minimizing functional redundancy across classification systems (e.g., avoiding overlap between functionally equivalent COGs and Pfams), (3) ensuring functional diversity by including FUs across broad functional categories, and (4) including FUs significant across all treatments as well as those specific to individual treatments. Altogether, a total of 61 FUs were selected for further analyses (Table 1) including two-component systems (TCSs), transcriptional regulators, transporters, components of the type I and II secretion systems (T1SS and T2SS, respectively), efflux pumps, cell protection systems, FUs related to catabolism of sugars and proteins, membrane/cell wall biosynthesis, and others involved in, e.g., phage integration, DNA replication, or motility.

**Table 1:** Selected functional units (FUs) enriched in fast-growers compared to slow-growers in the different treatments

Category	FU ID	Treatments	Short description	FU custom name	Acronym explanation
Two-component systems and transcriptional regulators	PF00072	All	Response regulator receiver domain	Response regulator	N/A
	PF00990,COG2199	All	GGDEF domain, diguanylate cyclase (c-di-GMP synthetase) or its enzymatically inactive variants	GGDEF	Glycine-glycine-aspartate-glutamate-phenylalanine
	PF02518	All	ATPase domain present in histidine kinases and other ATP-binding proteins	ATPase (HK)	N/A
	COG0583,PF03466,PF00126	All	DNA-binding transcriptional regulator, LysR family	LysR	Lysine regulator
	PF00512	All	His Kinase A (phospho-acceptor) domain (CheA)	CheA	Chemotaxis protein A
	COG3706	All	Two-component response regulator, PleD family	PleD	Ple = pleiotropy
	PF00563,COG2200	All	EAL domain	EAL	Glutamate-alanine-leucine
	COG2205	All	K + -sensing histidine kinase KdpD	KdpD	Potassium (K) dependent pump
	COG0745,PF00486	All	OmpR family DNA-binding response regulator	OmpR	Outer membrane protein regulator
	PF13426,PF08447	All	PAS domain	PAS	**Per** (circadian period protein)—**Amt** (Aryl hydrocarbon receptor nuclear translocator—**Sim** (gingle-minded protein)
	COG3279	CD,PL,PD,DL,VL	DNA-binding response regulator, LytR/AlgR family	LytR/AlgR	Lytic regulator/alginate regulator
	COG1846	PL,PD,DL,VL	DNA-binding transcriptional regulator, MarR family	MarR	Multiple antibiotic resistance regulator
	PF14559	PL,PD,DL,VL	Tetratricopeptide repeat	Tetratricopeptide	N/A
	COG0789	PL,PD,DL,VL	DNA-binding transcriptional regulator, MerR family	MerR	Mercury resistance operon regulatory protein
	COG2972	PL,PD,DL,VL	Sensor histidine kinase YesM	YesM	Unknown
	COG0640	PL,PD,DL,VL	DNA-binding transcriptional regulator, ArsR family	ArsR	Arsenic resistance regulator
	COG3920	DL,VL	Two-component sensor histidine kinase, HisKA and HATPase domains	Histidine kinase	N/A
Transporters	COG0834	All	ABC-type amino acids transpoter	ABC amino acids transp.	ABC = ATP-binding cassette
	PF00892	All	EamA-like transporter family	EamA	Efflux of antimicrobial molecules
	COG3203	All	Porin	Porin	N/A
	PF00924	All	Mechanosensitive ion channel	Mechanosensitive channel	N/A
	COG2885	CD,PL,PD,DL,VL	Outer membrane protein OmpA and related peptidoglycan-associated (lipo)proteins	OmpA-like	Outer membrane protein
	PF13505	PL,PD,DL,VL	Outer membrane protein beta-barrel domain	Beta-barrel	N/A
	COG1638, COG3090	PL,PD	TRAP-type C4-dicarboxylate transport system	TRAP	Transporter-Regulated-Associated-Periplasmic
	COG1123	PL,PD	ABC-type glutathione transport system ATPase component, contains duplicated ATPase domain	ABC glutathione transp.	ABC = ATP-binding cassette
	COG0659, PF00916	PL,PD	Sulfate permease or related transporter, MFS superfamily	MFS sulfate transp.	MFS = major facilitator superfamily
	PF00593	DL,VL	TonB dependent receptor	TonB receptor	Unknown
	PF07690	DL,VL	Major Facilitator Superfamily	MFS	Major facilitator superfamily
	COG4773	DL,VL	Outer membrane receptor for ferric components	Fe transport	N/A
	COG2814	VL	Predicted arabinose efflux permease, MFS family	Arabinose efflux	N/A
Secretion systems	PF13437	All	HlyD family secretion protein	HlyD (T1SS)	Hemolysin secretion protein D
	PF16576	CL,CD,PL,PD,DL	Barrel-sandwich domain of CusB or HlyD membrane-fusion	CusB/HlyD (T1SS)	CusB = copper (Cu) and silver, cation efflux system B; HlyD = Haemolysin secretion protein D
	COG3267	CL,PL,PD,DL,VL	Type II secretory pathway, component ExeA	ExeA (T2SS)	Extracellular enzyme secretion protein A
	PF07963	CL,DL,VL	Prokaryotic N-terminal methylation motif	Pilins or others	N/A
	COG2165	CL,DL,VL	Type II secretory pathway, pseudopilin PulG	PulG (T2SS)	Pullulanase secretion protein G
	PF00353	PL,PD,DL,VL	Haemolysin-type calcium-binding repeat	RTX (T1SS)	Repeat in toxin domain
	COG1538	DL,VL	Outer membrane protein TolC	TolC (T1SS)	Tolerance to colicin protein C
Efflux pumps	COG0845	CL,PL,PD,DL,VL	Multidrug efflux pump subunit AcrA (membrane fusion prot.)	AcrA	Acridine resistance protein A
	PF00873,COG0841	CL,PL,PD,DL,VL	Multidrug efflux system from AcrB/AcrD/AcrF family	AcrB/AcrD/AcrF	Acridine resistance protein B, D, F
	COG1566	PL,PD,DL,VL	Multidrug resistance efflux pump	Efflux pump	N/A
	PF02321	DL,VL	Outer membrane efflux protein	Efflux protein	N/A
Cell protection systems	COG1902	All	2,4-dienoyl-CoA reductase or related, OYE family	Oxidoreductase (OYE)	OYE = old yellow enzyme
	COG0494	All	8-oxo-dGTP pyrophosphatase MutT and related, NUDIX family	Pyrophosphohydrolase	N/A
	PF00903	CL,PL,PD,DL,VL	Glyoxalase/Bleomycin resistance protein/Dioxygenase superfamily	Glyoxalase	N/A
	COG0346	PL,PD,DL,VL	Catechol 2,3-dioxygenase and related proteins	Dioxygenase	N/A
	COG0625,K00799	DL,VL	Glutathione S-transferase	Glutathione S-transf	N/A
	PF07992	DL,VL	Pyridine nucleotide-disulphide oxidoreductase	Oxidoreductase	N/A
	PF00583	All	Acetyltransferase (GNAT) family	Alpha-amylase	Gcn5-related N-acetyltransferase
Catabolism	PF00128	CL,PL,PD,DL,VL	Alpha-amylase	Glycosidase	N/A
	COG0366	CL,PL,PD,DL,VL	Glycosidase	Amino acid oxidase	N/A
	COG0665	DL	Glycine/D-amino acid oxidase (deaminating)	Alcohol dehydrogenase	N/A
	PF08240	VL	Alcohol dehydrogenase GroES-like domain	UbiH	Ubiquinone biosynthesis protein H
Membrane/ wall synthesis	COG0654	PL,PD,DL,VL	2-polyprenyl-6-methoxyphenol hydroxylase and related	Transpeptidase ErfK/SrfK	Erytrhomycin/streptogramin resistance efflux pumps
	COG1376	DL,VL	Lipoprotein-anchoring transpeptidase ErfK/SrfK	Transpeptidase	N/A
	PF03734	VL	L,D-transpeptidase catalytic domain	Phage integrase	N/A
Others	PF00589	All	Phage integrase	GNAT	Gcn5-related N-acetyltransferases
	PF00270,PF00271, COG0513	CL,CD,PL,PD,DL	Helicases	Helicases	N/A
	COG3637	CD,PL,PD,DL,VL	Opacity protein and related surface antigens	Opacity protein	N/A
	PF08281,PF04542, COG1595,K03088	PL,PD,DL,VL	Sigma-70, regions 2 and 4	Sigma 70	N/A
	PF13403	PL,PD,DL,VL	Hint domain	Hint	Hedgehog/intein domain
	PF00460,PF06429	PL,PD,DL,VL	Flagella basal body rod proteins	Flagella base	N/A

Specific treatments where a given FU was enriched in fast-growers. Short descriptions are provided by the eggnog-mapper pipeline [63]. Custom names for all FUs and the explanations of the acronyms they encompass, if applicable, are also presented. Functional unit is here a word used to refer to COGs, Pfams, KOs, and CAZYs

**Fig. 5:**
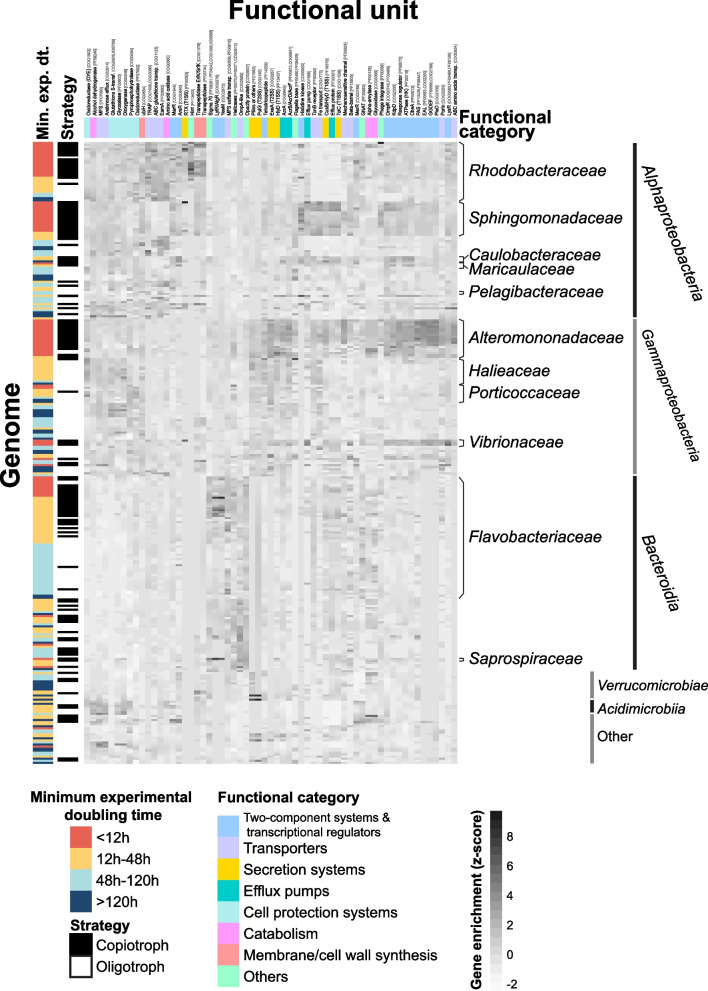
Clustered heatmap showing the distribution of selected functional units across bacterial groups. The most relevant families and classes are highlighted. Functional unit is here a word used to refer to COGs, Pfams, Kos, and CAZYs

We generated a heatmap to assess the taxonomic distribution of these 61 functional units (Fig. 5) and a table that displays the main fast-growing groups in which these FUs are enriched (Table 2).

**Table 2:**
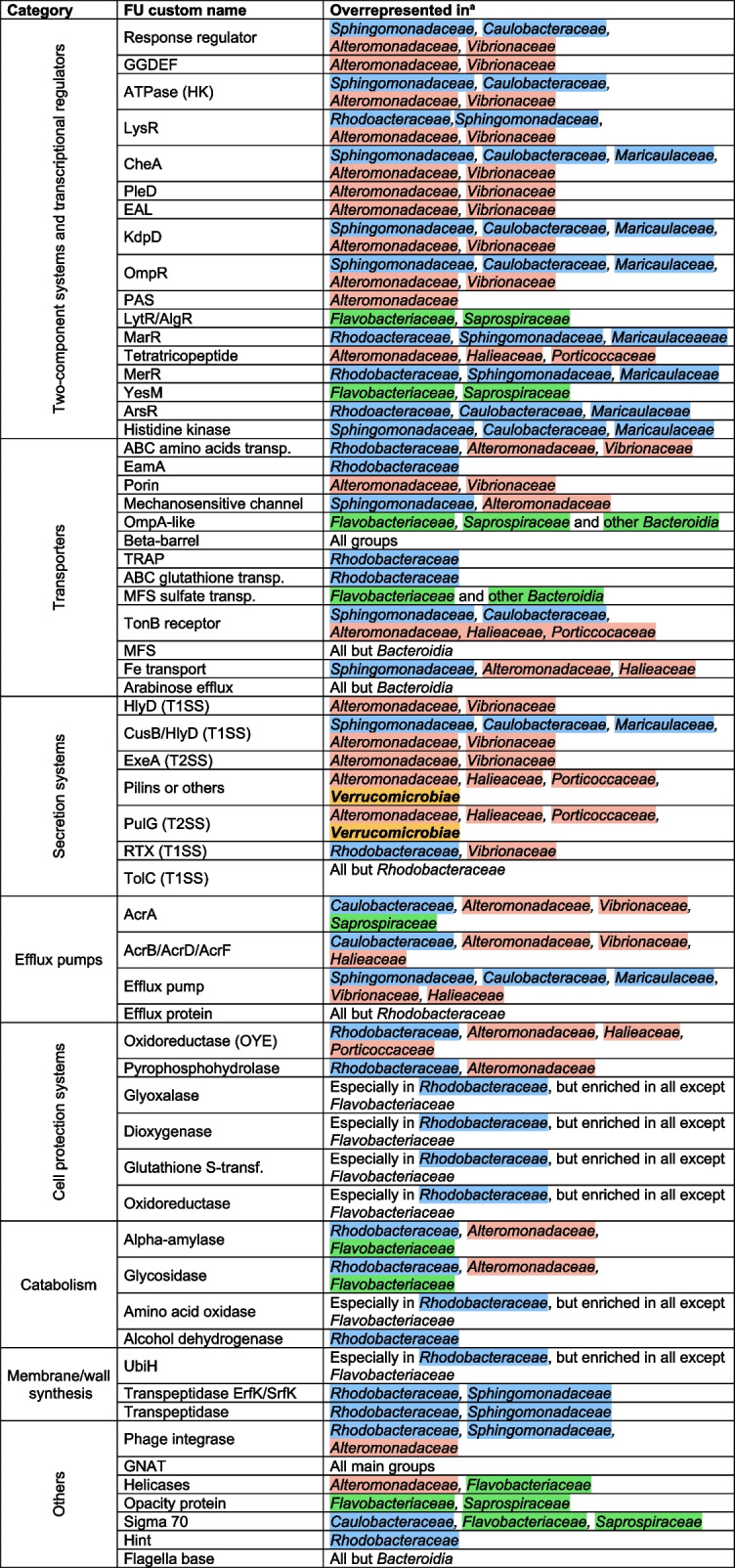
Distribution across the main bacterial groups of the selected functional units (FUs) enriched in fast-growers

^a^Taking into account the major groups (*Rhodobacteraceae, Sphingomonadaceae, Caulobacteraceae, Maricaulaceae, Alteromonadaceae, Vibrionaceae, Flavobacteriaceae,* and *Saprospiraceae*) with some additions when a given FU was especially enriched in another group (*Halieaceae, Porticoccaceae,* and *Verrucomicrobiae*). Some FUs were slightly overrepresented in more groups than indicated, we highlighted the most noteworthy ones to avoid overcomplication of the table

Custom names match those indicated in Table 1 and Fig. 5. Families are color-coded according to the class they affiliate with: *Alphaproteobacteria* = light blue, *Gammaproteobacteria* = salmon, *Bacteroidia* = green, *Verrucomicrobiae* = yellow. Functional unit is here a word used to refer to COGs, Pfams, Kos, and CAZYs

In general, all *Alphaproteobacteria* (including slow-growers) contained more amino acid oxidases, alcohol dehydrogenases, and certain transporters such as ATP-binding cassette (ABC) glutathione transporters or C4-dicarboxylate transporters; cell protection systems like dioxygenases or glyoxalases; and genes dedicated to ubiquinone synthesis (Fig. 5). The fast-growing members of this class were enriched in TCSs from the arsenic resistance (ArsR), multiple antibiotic resistance (MarR), and mercury resistance (MerR) families, characteristics that differentiate them from the other classes (Fig. 5, Table 2)

Within *Alphaproteobacteria*, the members of family *Rhodobacteraceae* that exhibited strong responses to the treatments were differentially characterized by an overrepresentation of domains like hedgehog/intein (Hint), repeat in toxin repeats (RTX), and transpeptidases (Fig. 5). Fast-growing genomic populations affiliated with the alphaproteobacterial families *Sphingomonadaceae*, *Caulobacteraceae*, and *Maricaulaceae* contained proportionally more FUs related to a particular histidine kinase (COG3920) that seems to be exclusive of these groups. Other characteristics that differentiated them from *Rhodobacteraceae* were the enrichment in an efflux pump, a Fe transporter, and a TonB receptor, traits that they often shared with *Gammaproteobateria*. Interestingly, these groups and, with less intensity, *Rhodobacteraceae*, were enriched in a series of TCSs also found overrepresented in gammaproteobacterial fast-growers, such as the chemotaxis protein A (CheA) and the K-dependent pump (KdpD; Fig. 5, Table 2).

Among the responsive *Gammaproteobacteria*, the families *Alteromonadaceae* and *Vibrionaceae* had the highest number of FUs related to two-component systems (Fig. 5), especially in domains like PAS, EAL, or GGDEF (see Table 1 for a breakdown of these acronyms) and a response regulator from the pleiotropy D (PleD) family. They also presented important numbers of components of the types I and II secretion systems efflux pumps. Additionally, they had noticeable amounts of an ABC amino acid transporter and a domain of mechanosensitive ion channels that were also enriched, though with less intensity, in some alphaproteobacterial fast-growers (Fig. 5, Table 2). *Vibrionaceae* differed from *Alteromonadaceae* by having a higher content of transcriptional regulators from the LysR family and a greater number of RTX domains. Other families that expressed fast growth within *Gammaproteobacteria*, such as *Halieaceae* and *Porticoccaceae*, did not contain many FUs related to TCSs, but they were enriched in pilins, a Fe transporter, a TonB receptor, tetratricopeptide repeats, and an oxidoreductase from the old yellow enzyme (OYE) family.

In general, genomes from class *Bacteroidia* (including slow-growers) were enriched in a sulfate transporter from the major facilitator superfamily (MFS), a porin from the OmpA (outer membrane protein A) family, and an opacity protein (Fig. 5). The genomes of fast-growing *Flavobacteriaceae* and *Saprospiraceae* were distinctively enriched in two-component systems from the lytic/alginate regulator (LytR/AlgR) and YesM families and had especially high quantities of the Sigma 70 factor and helicases (Fig. 5, Table 2).

### Functions conserved in slow-growers

The COG categories that were overrepresented in oligotrophs, such as translation, ribosomal structure and biogenesis (J category), energy production and conversion (C category), coenzyme transport and metabolism (H category), lipid and nucleotide transport and metabolism (I and F categories, respectively), and post-translational modifications, protein turnover and chaperones (O category) were overrepresented in the bacteria with the slowest growth in all the treatments that were not controls (except for the I category, which was only significant in the predator-reduced treatments; Fig. 4).

Among the FUs most enriched in slow-growers (Additional file 12: Table S3, bottom), several were associated with energy and metabolism, including acyl-CoA transferases, cytochrome components like the Rieske domain [91], and pyruvate/2-oxoglutarate dehydrogenase. Others were involved in protein synthesis and ribosome function, such as the 23S ribosomal subunit, proteins L23 and L29, and a ribosome-binding GTPase. Additionally, RNA-related elements like tRNA-associated proteins and anticodon-binding domains were notable, alongside cell division and maintenance proteins like the filamentation-temperature-sensitive protein (FtsL). Specialized enzymes enriched in slow-growers included glutamine amidotransferases, acetyl esterases, and sulfatases. Some FUs related to the transport of vitamins, Zn^+2^, Mg^+2^, Na^+^, ammonium, lipids, sugars, and biopolymers were also found enriched in slow-growers. Most of them were either MFS or ABC-type transporters.

### Biogeochemically relevant genes overrepresented in fast- and slow-growers

**Fig. 6:**
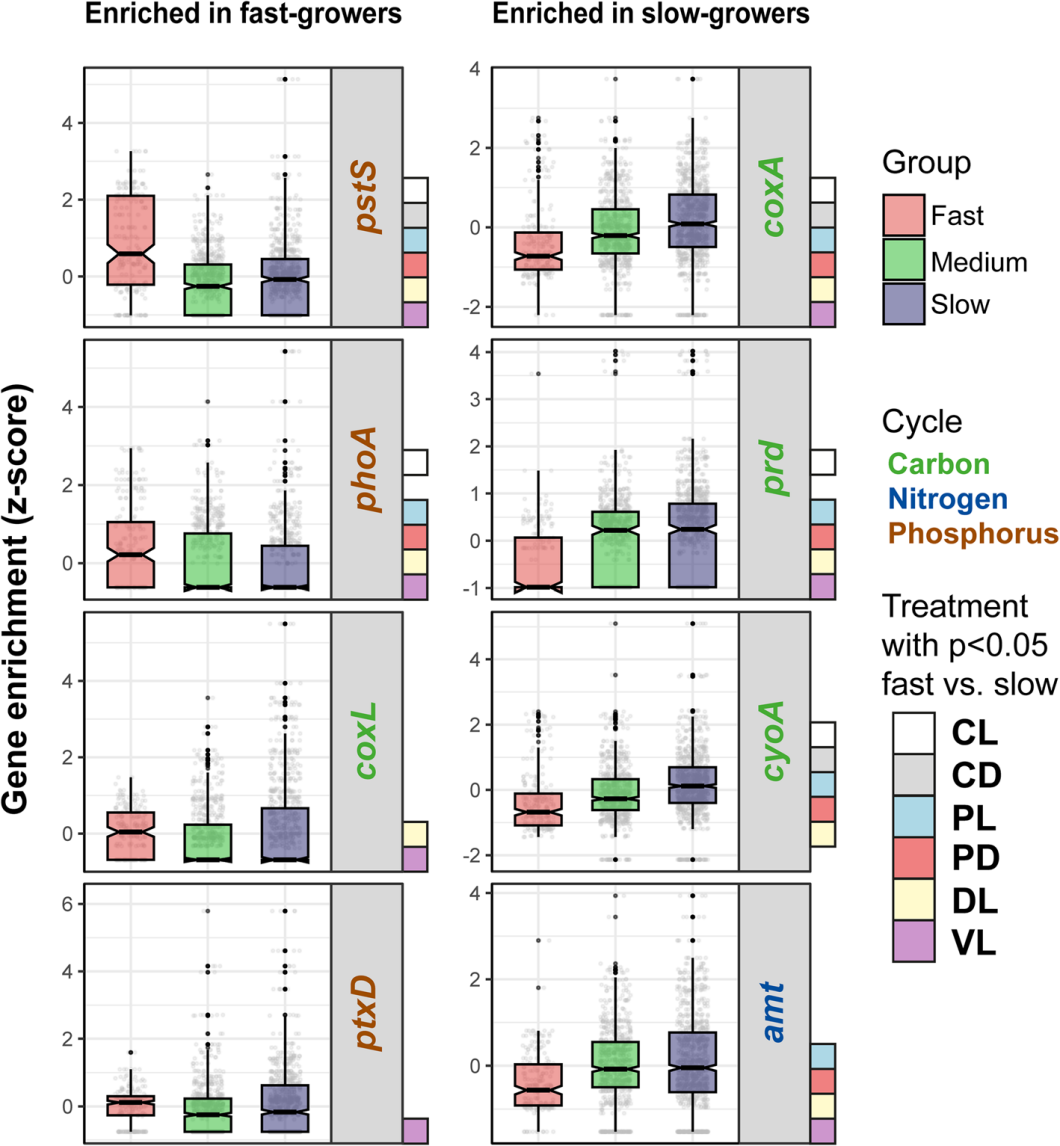
Main biogeochemically relevant genes enriched in fast-growers (red) or slow-growers (blue). Only those genes with clear overrepresentation (i.e., that they are clearly enriched in fast-growers compared to the medium- (green) and slow-growers) are displayed. The treatments with significant results (Wilcoxon rank sum test, *p* < 0.05) are indicated with colored squares. CL = control light, CD = control dark, PL = predator-reduced light, PD = predator-reduced dark, DL = diluted light, and VL = virus-reduced light

We tested the over- or underrepresentation of a selection of 49 genes with known relevant roles in ocean biogeochemical cycles across the genomes of this study (Additional file 13: Table S4). Six of them (*pstS*, *phoA*, *coxL*, *ptxD, pit,* and *gdhA*) were enriched in fast-growers compared to slow-growers in at least one treatment, and the other six (*coxA*, *prd*, *cyoA*, *amt*, *phoD*, and *pufM*) were overrepresented in slow-growers (Additional file 6: Figure S6). The genes *pstS* and *phoA* encoding a general phosphate transport system and alkaline phosphatase A, respectively, were the most prominently overrepresented in responsive bacteria across all treatments, although *phoA* was not significant in the CD experiment (Fig. 6). Genes *coxL* (CO dehydrogenase) and *ptxD* (phosphite dehydrogenase) were more abundant in fast-growers in all treatments, although this trend was significant only in DL (only *coxL*) and VL (both genes) treatments. On the other hand, *ghdA* (glutamate dehydrogenase) and *pit* (low-affinity inorganic phosphate transporter) were not specific to or responsive to bacteria since they appeared more abundant in medium-growers (Additional file 6: Figure S6).

Among the genes enriched in slow-growers, *coxA* (cytochrome c oxidase subunit I), *prd* (proteorhodopsin), and *cyoA* (cytochrome o ubiquinol oxidase subunit II) were clearly overrepresented in almost all treatments (Fig. 6). The gene *amt* (ammonium transporter) was significantly enriched in all treatments except for the controls (Fig. 6). The gene *pufM*, although having a significant enrichment in slow-growers (Wilcoxon rank sum test, *p* < 0.001) was too rare to draw valid conclusions (Additional file 6: Figure S6) and *phoD* (alkaline phosphatase D) was significantly enriched only in slow-growers of the CD treatment (Additional file 6: Figure S6).

### Differential traits of bacterial bloomers

We defined “bloomers” here as those populations that had an FEDT lower than 12 h and grew to represent more than 1% of the total reads in the same experiment. This is a more restrictive term than “fast-growers” or “responsive bacteria” (used as synonyms in this study), which only took into account the FEDT and not the abundance. We tested whether bacterial blooms occurred in the treatments and identified 18 genomic populations in our dataset that could be considered to have this behavior. These populations were affiliated with families *Alteromonadaceae*, *Vibrionaceae*, *Rhodobacteraceae*, and *Flavobacteriaceae* (Additional file 7: Figure S7, Additional file 8: Figure S8, and Additional file 10: Table S1).

**Fig. 7:**
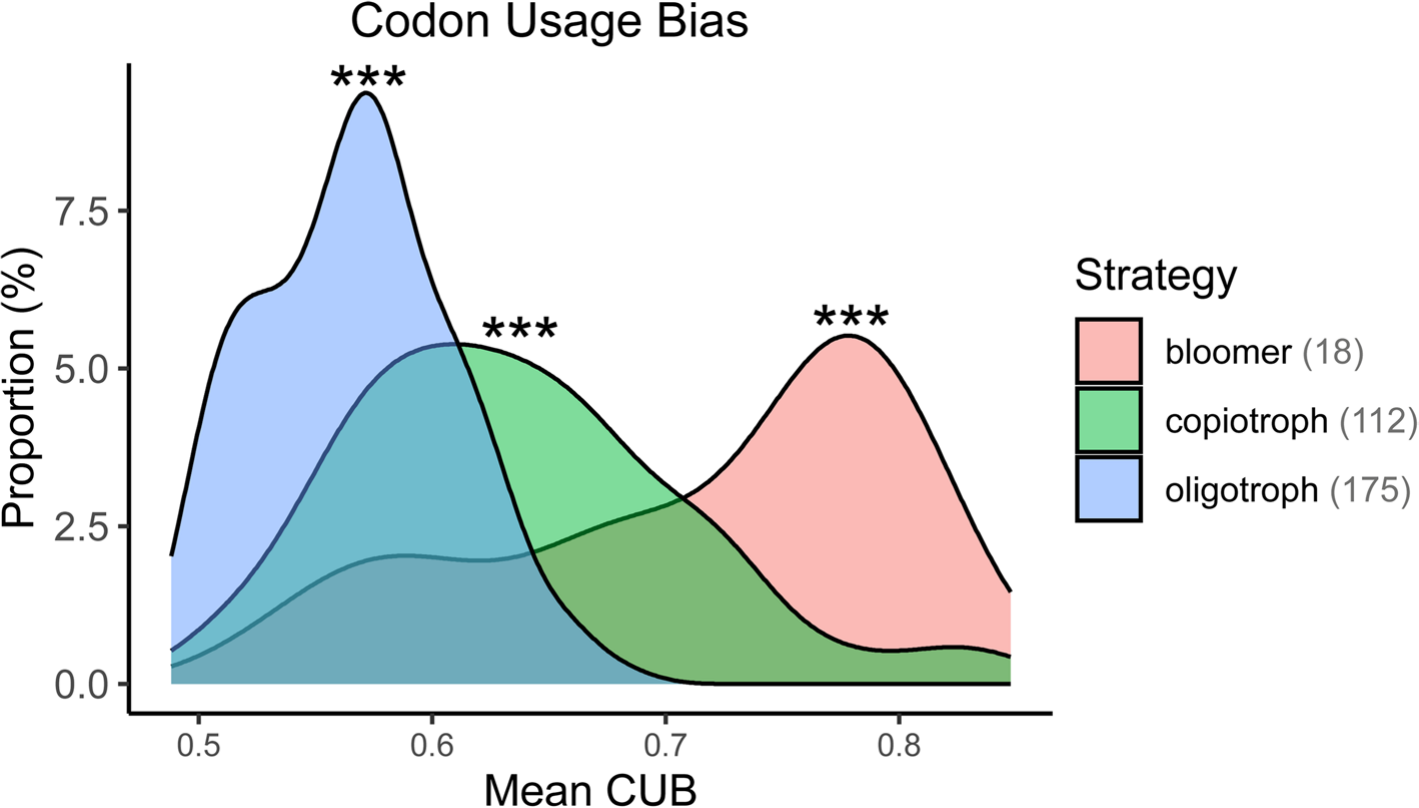
Difference in codon usage bias between bloomers, copiotrophs, and oligotrophs in this study. The number of genomes in each group is indicated in the legend. *** = Pairwise Wilcoxon rank sum test, *p* < 0.001

Among the genomic traits analyzed, neither the G + C content nor the genome size was significantly different between bloomers and the rest of the copiotrophs (Additional file 9: Figure S9A, B). However, the EMDT (Additional file 9: Figure S9C) and, more clearly, CUB (Fig. 7), were significantly higher in the bloomers. The COG categories S (unknown), T (signal transduction mechanisms), and K (transcription) were overrepresented in bloomers compared to the rest of the copiotrophs, mirroring the pattern observed in fast-growers relative to slow-growers. In total, there were 149 functional units enriched in bloomers compared to the rest of the copiotrophs (Additional file 14: Table S5), but most of them coincided with the ones already seen as overrepresented in the genomes classified as fast-growers in the treatments.

## Discussion

This study shows that certain bacterial representatives from a broad taxonomic set of families are able to respond to experimental manipulations with fast growth. This is consistent with previous experimental work and field studies on the ecological strategies of marine bacteria in general and of copiotrophic bacteria in particular [31, 35, 42, 92–94]. The experimental findings of this study could be useful to predict the behavior of marine bacteria in real-world scenarios where nutrient availability increases, such as phytoplankton blooms or inputs of Saharan dust or wildfire ashes, which have been predicted to become more frequent in the future [95–98]. Additionally, rising UV radiation due to the climate crisis is expected to reduce viral activity, a phenomenon also relevant to the current experiments [99].

We acknowledge, though, that the thresholds set in this work to define fast-growers and bloomers are relatively narrow and partially arbitrary, and thus, we might be missing information from relevant bacteria that were not considered in these categories. We also note that the fold-change–based experimental doubling times that we work with were inferred from only two timepoints. Thus, we might not have captured the growth potential of bacteria that could have grown in the early phase of the experiments but declined before the end, or those that might have started to grow near the final time. The use of relative abundances implies that the growth observed in the experiments could have been, in some cases, just a change in rank order within the community and not in absolute abundance, and this is another limitation of our study. However, given the increase in total bacterial abundance observed in the flow cytometry data, we are relatively confident that the bacteria that increased their relative abundance also increased in number.

### Responsive bacteria are enriched in specific genes

Importantly, our analyses uncovered several functions that could contribute to explain the ability of some bacteria to respond to the manipulations. Through our comparative genomic analyses, we identified a series of individual functional units (FUs) that were especially related to fast growth in the treatments and showed that they had a differential enrichment across the responsive bacteria.

One of the main groups of FUs observed here to be related to fast growth were the two-component systems (TCSs). TCSs are mechanisms formed by a protein with a sensory domain that detects specific stimuli and a histidine kinase domain that activates the other component, which is a transcriptional regulatory protein called a response regulator [100]. TCSs are the most common mechanisms used by bacteria to sense changes in the environment and react to them [101], and thus, it is not unexpected to find them as the most important mechanism explaining fast growth in our study, as it has also been in previous ones [100]. The FUs related to TCSs overrepresented in the genomes of fast-growers in this study participate in the regulation of adhesion, biofilm formation, quorum sensing, cell growth, motility, osmoregulation, and detoxification (Additional file 15: Table S6), common mechanisms in copiotrophic and opportunistic bacteria [19–21, 102]. For example, CheA is a histidine kinase involved in modifying the swimming behavior of the flagella in response to stimuli such as the presence of nutrients [103], and GGDEF is a domain of diguanilate cyclase present in several TCSs that sense oxygen and changes in light and regulate biofilm formation, among other functions [104]. TCSs have been related to marine biogeochemical cycling [100], so their overrepresentation in fast-growers may imply that, in the event of a bloom, these bacteria may have a substantially larger influence on elemental balances in the ocean than expected from their average abundances.

Our results evidenced that fast-growers were enriched with a set of transporters that not only would help them to acquire small or large molecules for nutritional purposes, but would also be involved in osmoregulation, competition, adhesion, and biofilm formation (Additional file 15: Table S6). For example, the mechanosensitive channels that were overrepresented in the genomes of responsive bacteria are pores that gate in response to mechanical tension, allowing ions to circulate and equilibrate osmotic pressure, and have a role in maintaining K^+^ homeostasis [105], which seems to be a key mechanism for fast growth. The outer membrane protein A (OmpA-like) domain has been found in proteins related to flagellar motility [106] or biofilm formation [107], and the arabinose efflux permease could be related to competition with other bacteria [108] or to symbiotic relationships with algae [109].

Structural components of the type I and II secretion systems, and proteins that are secreted by them, were also enriched in responsive bacteria. Both systems are involved in secreting a wide range of proteins to interact with other cells, degrade external compounds, or colonize surfaces or hosts [110, 111]. As they facilitate cell attachment, they are key to establish associations with eukaryotes [112] and resist predation and viral lysis [113, 114]. Importantly, it has been stated that they could even affect biogeochemical cycling in the oceans [112]. Interestingly, pilins and pseudopilins, which act like pistons in protein secretion through T2SS [111], were greatly enriched in two populations of *Verrucomicrobiae* that were predicted to be oligotrophs and did not react to the treatments. It is known that some members of *Verrucomicrobiae* are enriched in components of the pilus apparatus, which play an important role in key aspects of their ecology such as twitching motility, surface attachment, and host colonization [115–117], but the number of genomes from this class in our study was too low to statistically prove if these components were more enriched in fast-growing members than in the slower ones.

The genomes of fast-growers in our experiments contained high numbers of efflux pumps, membrane proteins widely spread through bacteria that export toxic substances like antibiotics or waste metabolism byproducts [118]. Although they have been traditionally involved in antibiotic resistance and pathogenicity, they have also been linked to biofilm formation and quorum sensing [119], so the possibility that they participate in fast growth is reasonable.

Protection systems against metabolites with negative influence on growth were identified as relevant components of the genomic repertoire of responsive bacteria, especially in the case of the *Rhodobacteraceae*. Examples of this are glyoxalases, which detoxify reactive aldehydes like methylglyoxal that are commonly produced in metabolism [120], or glutathione S-transferases, which catalyze the conjugation of glutathione with xenobiotic compounds and protect against oxidative stress and antimicrobials [121]. Additionally, some of the efflux pumps and TCSs that were enriched in fast-growers regulate the defense against oxidative stress, antimicrobials, toxins, and osmotic pressure (Additional file 15: Table S6), stressing the importance of protection in fast growth. The high production of toxic byproducts in fast-growing bacteria, driven by their active metabolism, underscores the importance of mechanisms that mitigate toxic compounds and metabolites, as well as their regulation, for sustaining rapid growth.

Responsive bacteria (especially *Rhodobacteraceae*) were also enriched in genes related to membrane/cell wall biosynthesis and peptidoglycan crosslinking, a necessary step for cell wall assembly [122]. These results are logical, since membranes need to be created fast when a cell divides frequently. Moreover, bacterial cell walls offer protection against toxic compounds and osmotic pressure, and they facilitate cell adhesion, which are considered important processes for the survival of fast-growers [123].

Altogether, our results and their putative ecological meaning (Additional file 15: Table S6) suggest that fast-growing bacteria in marine environments are specialized in resisting several types of stresses (osmotic pressure, predation, low nutrients, and toxins) by neutralizing harmful substances, associating (symbiotically or not) to other organisms, attaching to surfaces, and/or forming biofilms. They seem to be prepared to scan their surroundings through their highly present TCSs, and when they detect nutrients, they may move toward them and activate all their expensive metabolic machinery to acquire and assimilate them at any price. When conditions are ideal, they appear to be well prepared to grow and divide fast (implying high metabolic rates, which likely produce toxic by-products, which they are specialized in neutralizing), and thus, some of them can bloom. These traits align with the classical r/K-selection framework, where fast-growing bacteria exhibit *r*-strategist characteristics: under favorable conditions, they express rapid reproduction, high metabolic activity, and opportunistic resource exploitation. However, under stress, their focus shifts from growth to survival mechanisms and environment scanning, enabling persistence at low population densities [124].

### Bloomers differ from the rest of the copiotrophs

Aside from the functional characterization of fast-growing bacteria, we aimed to determine whether any genomic or functional trend differentiated bacteria that not only grow rapidly but that can also reach abundant proportions within the community (herein referred to as “bloomers”). This would contribute to answering the fundamental question: “Can any copiotroph bloom, or are only certain ones capable of doing so?”. Bloomers (those genomes that, at least once, reached 1% relative abundance or more and had an experimental doubling time of less than 12 h) differentiated from the rest of copiotrophs in their codon usage bias (Fig. 7, Additional file 9: Figure S9), which suggests that they have a higher tendency among copiotrophs to select ribosomal proteins that translate more efficiently. These results should be interpreted with caution, as the thresholds used to classify bloomers are somewhat arbitrary, and other copiotrophs not identified here may bloom under different experimental conditions than the ones tested in this study. Nonetheless, the FUs found to be enriched in bloomers (Additional file 14:Table S5) could be used as markers for heterotrophic bacteria that could possibly exhibit this blooming behavior in optimal conditions for their growth.

### Bacterial blooms could impact biogeochemical cycles

Within the biogeochemically relevant genes that were enriched in fast-growers, four of them (*pstS*, *phoA*, *pit*, and *ptxD*) were related to the phosphorus cycle, one (*coxL*) to the carbon cycle, and one (*gdhA*) to the nitrogen cycle. From these, the ones clearly overrepresented in fast-growers were *pstS*, *phoA*, *coxL*, and *ptxD* (Fig. 6). The *coxL* gene encodes the large subunit of the CO dehydrogenase and confers marine bacteria the capacity of obtaining energy through the oxidation of CO, a mechanism that enhances long-term survival in oligotrophic conditions [125]. These data align with the earlier observation that fast-growers are enriched in stress resistance genes and reinforce the idea that they are especially prepared to survive until conditions for fast growth are met. This capacity is especially relevant in oligotrophic environments such as the Mediterranean Sea (where our experiments were conducted), an environment mostly limited by phosphorus [126]. While *pstS* encodes for a protein related to a phosphate transport system [127], *phoA* is the alkaline phosphatase A, which catalyzes the oxidation of phosphate [128], and *ptxD* encodes for a phosphite dehydrogenase [129]. Given the limiting nature of phosphorus in our site of study, it is possible that genes related to its cycle are especially relevant for fast-growers in this environment. It should be noted, though, that the differences described in this section are not absolute, since the groups show certain overlap (Additional file 6: Figure S6). Nonetheless, the results are statistically significant: it it is likely that certain biogeochemical cycles would be impacted in the event of a bacterial bloom. The capacity for blooming could be strongly influenced by the ability to sense and acquire phosphate.

### Slow-growers keep essential functions

It is known that genes representing essential processes tend to be overrepresented (or enriched) in the genomes of oligotrophs, as they are streamlined and have lost everything that is “optional” [8]. This observation correlates with the results obtained in this study. Several of the FUs enriched in slow-growers (Additional file 12: Table S3) were essential for ribosome function and translation. Ribosome-binding GTPases play crucial roles in the assembly, function, and regulation of ribosomes during protein synthesis [130]; the anticodon-binding domain is a structural feature of tRNA synthetases [131], and peroxiredoxins are involved in post-translational modifications [132]. Other enzymes, such as glutamine amidotransferases, play an essential role in the biosynthesis of amino acids, nucleotides, or coenzymes [133]. The Rieske domain, also enriched in slow-growers, is a component of cytochromes, which are components of the electron transport chain, which facilitates ATP synthesis [91]. This group of bacteria was also rich in pyruvate/2-oxoglutarate dehydrogenases, which participate in the tricarboxylic acid cycle [78], and in acetyl esterase/lipase, which is involved in lipid degradation [78]. In addition, CoA transferases, which were also frequent in slow-growers, are involved in a wide range of processes such as the tricarboxylic acid cycle, energy production, and amino acid and lipid metabolism [78]. Another protein enriched in slow-growers was FtsL, a part of the divisome complex that coordinates cell wall synthesis during cell division [134]. Our results agree with existing literature that reports that oligotrophs, or bacteria that grow slowly, are enriched in genes related to lipid [7], amino acid, [102], nucleotide [102, 135], coenzyme metabolism [17], energy processes [17, 20], or post-translational modifications [20, 136]. Contrary to our findings (Fig. 4), the COG category J (translation, ribosomal structure, and biogenesis) has never been reported, to our knowledge, to be enriched in oligotrophs or slow-growers. Nonetheless, our data correlate with the evolutionary explanation of trophic strategies as the FUs that account for the enrichment of the translation category are structural ribosomal proteins essential for bacterial functioning.

### Fast- and slow-growers have different transport strategies

In this study, we have shown that, while fast-grower genomes contained a large variety of transporters, including both high- and low-affinity systems, slow-growers were enriched in fewer transporters, primarily high-affinity ones. Copiotrophs are usually shown to have higher numbers of transporters than oligotrophs [17, 20, 102], in agreement with what we observed. However, several studies have shown oligotrophs to be specialized in high-affinity transport, while copiotrophs would be using especially low-affinity systems [7, 22, 137]. Although our analysis does not contradict the observation that oligotrophs use mostly high-affinity transporters, it shows that fast-growers also rely significantly on these transporters, as hinted at in a previous study [138]. What appeared different between both groups in our results was the types of molecules that they transported: high-affinity transporters of fast-growers channeled phosphate, glutathione, K +, or amino acids, while slow-growers transported lipids, vitamins, divalent cations, ammonium, or sugars. This could indicate that fast-growers are more likely to invest in high-affinity systems for molecules that have a direct impact on rapid growth, while slow-growers would prioritize molecules that support metabolic maintenance and are less energetically costly to transport.

## Conclusions

Here, we have functionally characterized marine bacteria that respond with rapid growth to environmental change, identifying indicator genomic features across different taxonomic groups and providing a list of genes that could serve as potential markers for bloomers in future research. Additionally, our findings provide hints that suggest that among copiotrophs, certain heterotrophic bacteria may be better equipped than others to bloom, though this requires further investigation. We also provide hints on how heterotrophic bacterial blooms could influence biogeochemical cycling, particularly the phosphorus cycle in the studied system. To our knowledge, this is the first study that delves into the functional characterization of bacterial blooming events based on experimental data. While marine heterotrophic bacterial blooms have been understudied in comparison to, e.g., algal blooms, our work sheds light on the microbial functions associated with these blooms and emphasizes their ecological importance. This work sets the stage for future experiments (e.g., targeted knockouts, single-cell physiological measurements, or time-resolved growth curves), which could confirm how these traits directly shape bloom success.

## Supplementary Information

Additional file 1: Figure S1. Correlation between the estimated and experimental minimum doubling times. Estimated minimum doubling times (EMDTs) were computed with the gRodon R script, and experimental minimum doubling times were based in the fold-change of the genome abundances during the experiments as explained in the methods section. The r squared was calculated using the base R linear model function

Additional file 2: Figure S2. Flow cytometry abundances of the whole community during each treatment of the experiments. The x-axis is the time of the experiment in hours, and the y-axis is the exponential value of the total quantity of cells per milliliter as obtained by flow cytometry. The fall season is included as complementary information, although it is not considered in this study

Additional file 3: Figure S3. A. Percentages of the total reads in the metagenomes represented by our genomes in every treatment and time averaged across seasons. B. Relative portion of metagenomic reads representing genomes predicted as oligotrophs or as copiotrophs by gRodon [17] in every treatment and time averaged across seasons. CL = control light, CD = control dark, PL = predator-reduced light, PD = predator-reduced dark, DL = diluted light, VL = virus-reduced light, t0 = initial time, tf = final time

Additional file 4: Figure S4. Response of the most prevalent bacterial families in this study to the different treatments. Distribution of fold-change based experimental doubling times (FEDTs) across the different families and treatments. The y-axis is the frequency of each FEDT (x-axis), therefore the peaks represent those FEDTs which were most frequent in each family and treatment. The number of data points in each plot is indicated in blue. CL = control light, CD = control dark, PL = predator-reduced light, PD = predator-reduced dark, DL = diluted light, VL = virus-reduced light

Additional file 5: Figure S5. Proportion of fast-, medium- and slow-growers and their distribution by class in each treatment. The number of genomic populations that are fast-growers in each treatment is indicated. CL = control light, CD = control dark, PL = predator-reduced light, PD = predator-reduced dark, DL = diluted light, VL = virus-reduced light

Additional file 6: Figure S6. All biogeochemically relevant genes enriched in fast-growers (red) or slow-growers (blue) and their variation across treatments. The grey boxes indicate those genes that have not been significantly enriched (Wilcoxon Rank Sum test p < 0.05). CL = control light, CD = control dark, PL = predator-reduced light, PD = predator-reduced dark, DL = diluted light, VL = virus-reduced light

Additional file 7: Figure S7. Relationship between doubling times and relative abundance of each genome at the final time of the experiments. Those which reached fold-change based experimental doubling times of > 12 h and final relative abundances of > 1% were classified as bloomers. A pie chart outlines the family distribution of these bloomers

Additional file 8: Figure S8. Abundances of bloomers across treatments and seasons. Relative abundances in the initial (t0) and final times (tf; 36 h in winter and summer, 48 h in spring) of the genomes designated as bloomers in this study in each season and treatment. The Genome IDs and their GTDB taxonomic classification up to the highest level without placeholders, are also indicated. f = family; g = genus; s = species; CL = control light; CD = control dark; PL = predator-reduced light; PD = predator-reduced dark; DL = diluted light; VL = virus-reduced light

Additional file 9: Figure S9. Comparison of the genomic properties of bloomers, copiotrophs and oligotrophs.A. Genome sizes across strategies presented as boxplots. B. G + C content (%) across strategies. C. Density plots of estimated minimum doubling times (EMDTs) across strategies. D. Density plots of codon usage bias (CUB) across strategies. *** = Pairwise Wilcoxon Rank Sum test p < 0.001

Additional file 10:Table S1. Quality, genomic data, taxonomic classification (GTDB), trophic strategy and behavior of the genomes analyzed in the different treatments of this work. FEDT = fold-change based experimental doubling time; CL = control light; CD = control dark; PL = predator-reduced light; PD = predator-reduced dark; DL = diluted light; VL = virus-reduced light

Additional file 11: Table S2. Abundances and doubling times of all genomic populations across treatments and seasons. t0 = initial time; tf = final time

Additional file 12: Table S3. Functional units enriched in fast-growing bacteria (Wilcoxon Rank Sum test p < 0.05) ordered by the difference in the mean proportion of genes from each functional unit between fast- and slow-growers

Additional file 13: Table S4. Biogeochemically relevant genes screened in this study and their references

Additional file 14: Table S5. Functional units enriched in bloomers (Wilcoxon Rank Sum test p < 0.05) ordered by the difference in the mean proportion of genes from each functional unit between bloomers and copiotrophs

Additional file 15: Table S6. Ecological functions of the selected functional units (FUs) enriched in fast-growers according to the literature. Functional unit is here a word used to refer to COGs, Pfams, KOs and CAZYs

## Data Availability

The metagenomes used in this study are publicly available in the European Nucleotide Archive and NCBI under BioProject PRJEB64576. The MAGs and isolate genomes are available in NCBI under BioProjects PRJNA1073303 and PRJNA1068984, respectively. Amplicon sequencing data of the V4-V5 region of the 16S rRNA gene used to select the isolates for whole-genome sequencing are publicly available in the European Nucleotide Archive under BioProject PRJEB60085.
