# Supplementary figures and images for: Decoding the genetic drivers of marine bacterial blooms through comparative genomics
Source: Microbiome. 2025 Oct 1;13:198. doi: 10.1186/s40168-025-02182-y (PMC12487129; doi:10.1186/s40168-025-02182-y)

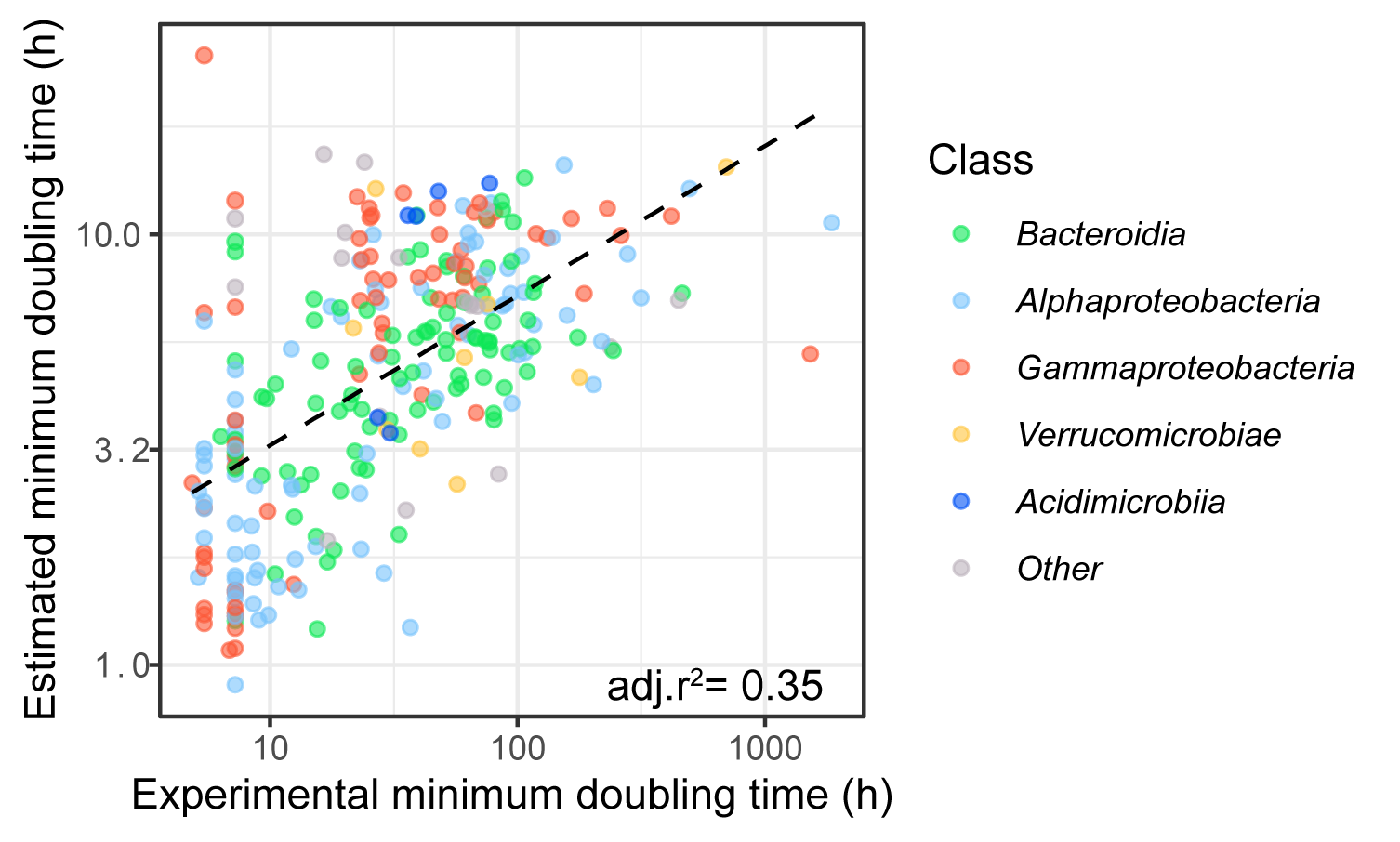

Supplement: Supplementary file 2 — Additional file 1: Figure S1. Correlation between the estimated and experimental minimum doubling times. Estimated minimum doubling times (EMDTs) were computed with the gRodon R script, and experimental minimum doubling times were based in the fold-change of the genome abundances during the experiments as explained in the methods section. The r squared was calculated using the base R linear model function [file 40168_2025_2182_MOESM1_ESM.tif]

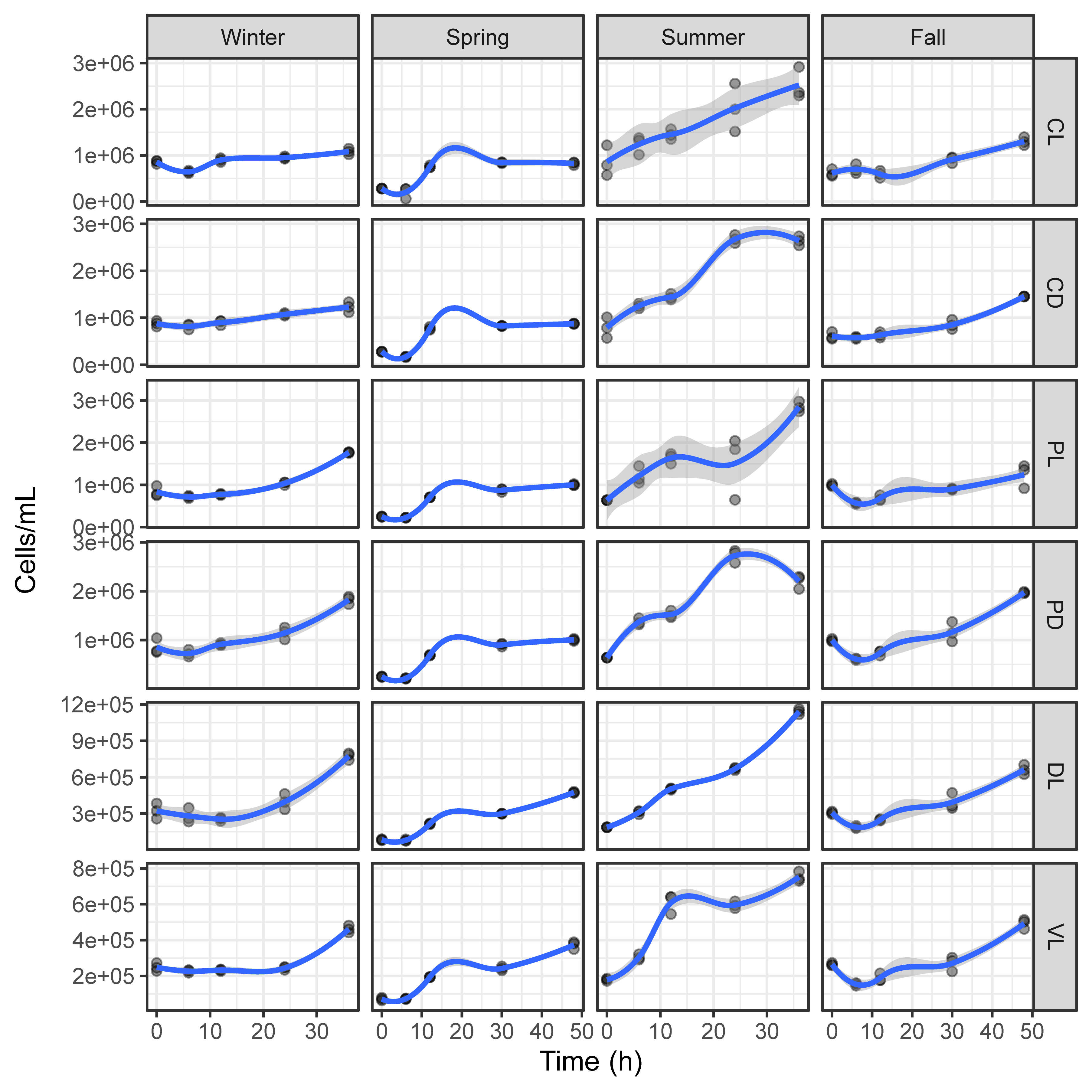

Supplement: Supplementary file 3 — Additional file 2: Figure S2. Flow cytometry abundances of the whole community during each treatment of the experiments. The x-axis is the time of the experiment in hours, and the y-axis is the exponential value of the total quantity of cells per milliliter as obtained by flow cytometry. The fall season is included as complementary information, although it is not considered in this study [file 40168_2025_2182_MOESM2_ESM.tif]

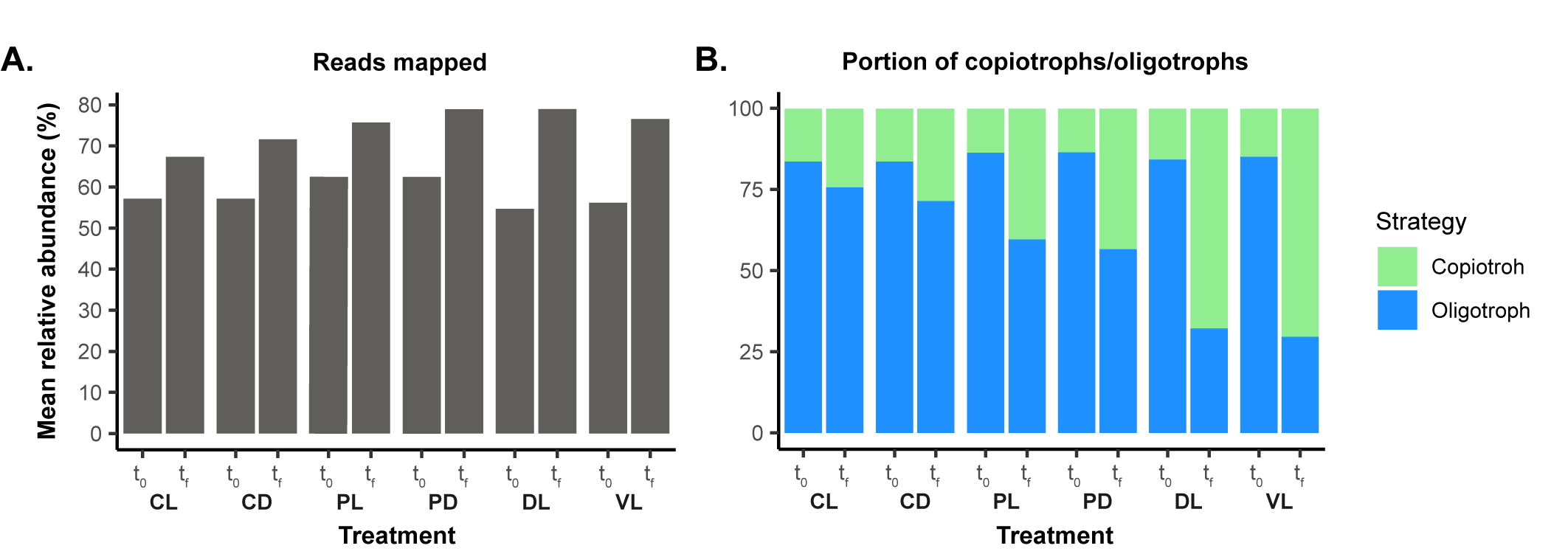

Supplement: Supplementary file 4 — Additional file 3: Figure S3. A. Percentages of the total reads in the metagenomes represented by our genomes in every treatment and time averaged across seasons. B. Relative portion of metagenomic reads representing genomes predicted as oligotrophs or as copiotrophs by gRodon [17] in every treatment and time averaged across seasons. CL = control light, CD = control dark, PL = predator-reduced light, PD = predator-reduced dark, DL = diluted light, VL = virus-reduced light, t0 = initial time, tf = final time [file 40168_2025_2182_MOESM3_ESM.tif]

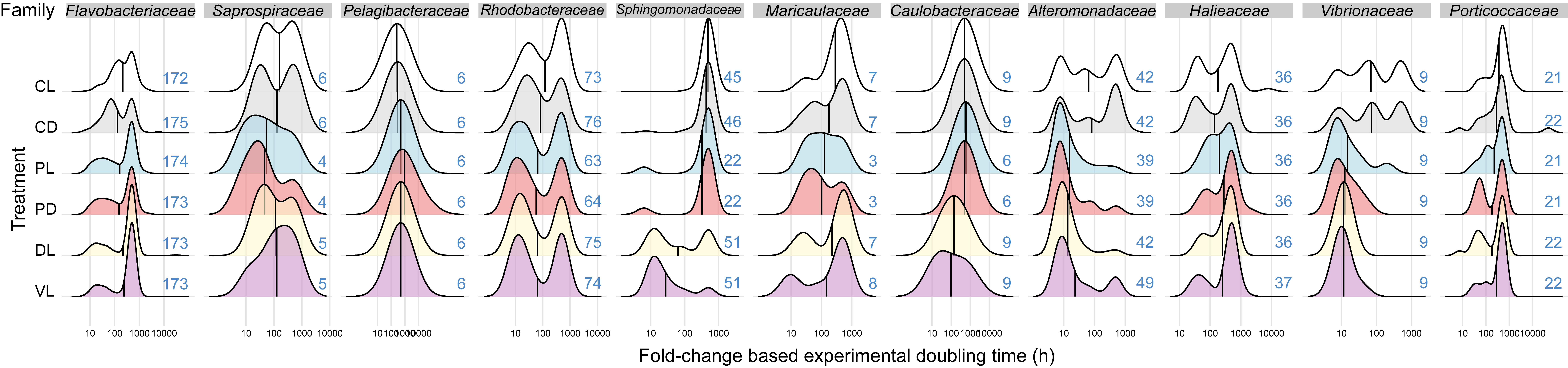

Supplement: Supplementary file 5 — Additional file 4: Figure S4. Response of the most prevalent bacterial families in this study to the different treatments. Distribution of fold-change based experimental doubling times (FEDTs) across the different families and treatments. The y-axis is the frequency of each FEDT (x-axis), therefore the peaks represent those FEDTs which were most frequent in each family and treatment. The number of data points in each plot is indicated in blue. CL = control light, CD = control dark, PL = predator-reduced light, PD = predator-reduced dark, DL = diluted light, VL = virus-reduced light [file 40168_2025_2182_MOESM4_ESM.tif]

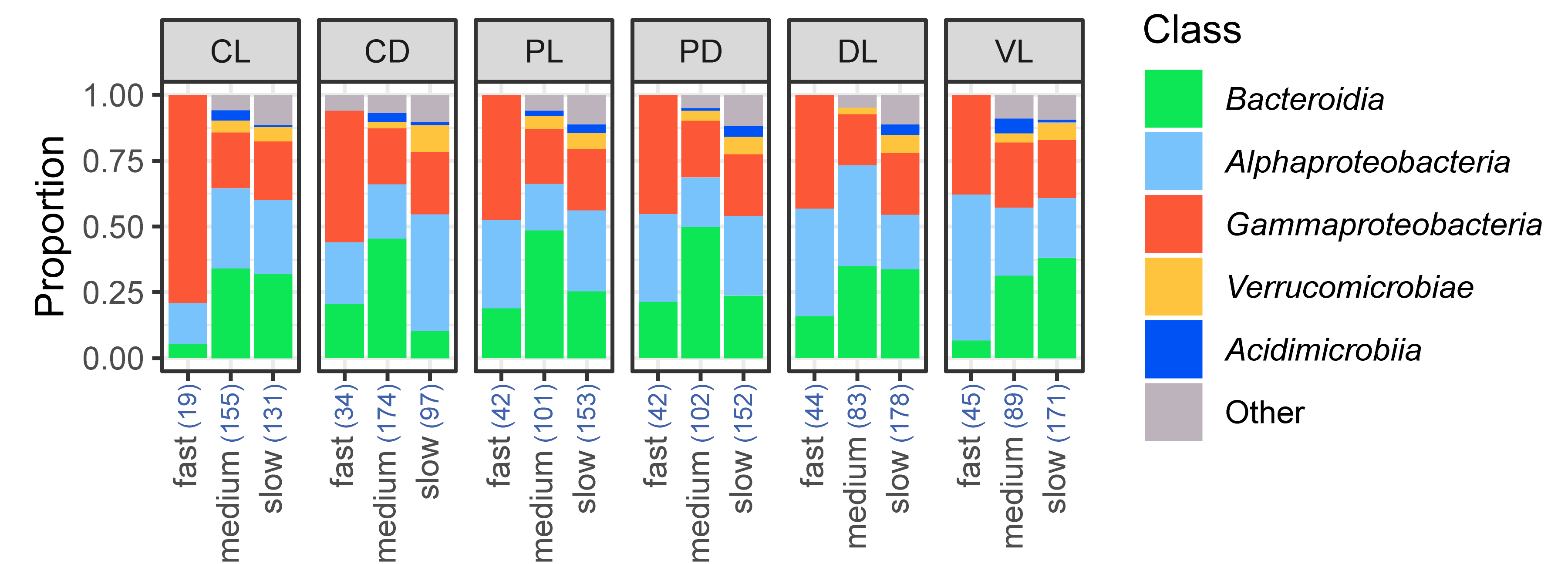

Supplement: Supplementary file 6 — Additional file 5: Figure S5. Proportion of fast-, medium- and slow-growers and their distribution by class in each treatment. The number of genomic populations that are fast-growers in each treatment is indicated. CL = control light, CD = control dark, PL = predator-reduced light, PD = predator-reduced dark, DL = diluted light, VL = virus-reduced light [file 40168_2025_2182_MOESM5_ESM.tif]

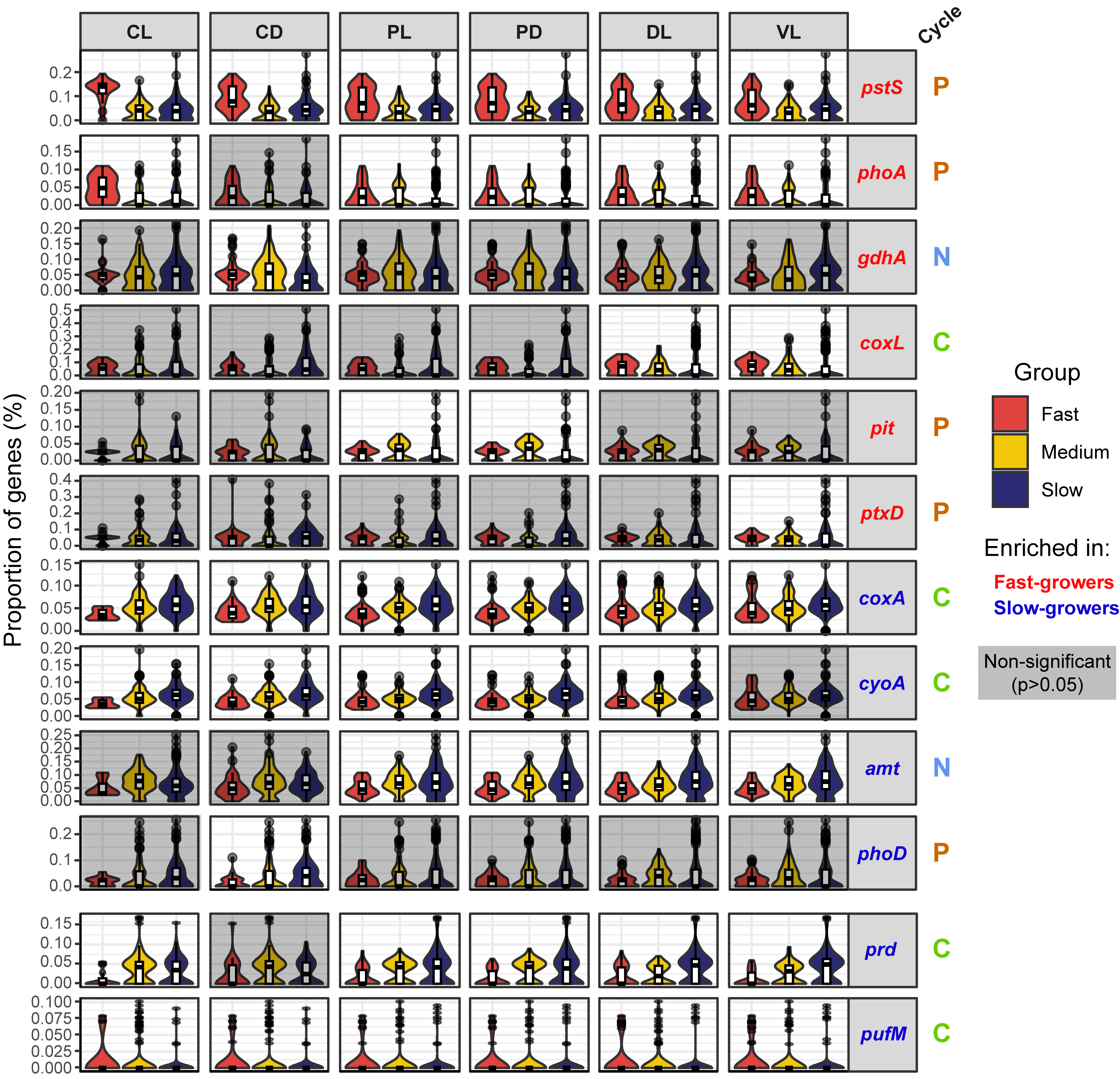

Supplement: Supplementary file 7 — Additional file 6: Figure S6. All biogeochemically relevant genes enriched in fast-growers (red) or slow-growers (blue) and their variation across treatments. The grey boxes indicate those genes that have not been significantly enriched (Wilcoxon Rank Sum test p < 0.05). CL = control light, CD = control dark, PL = predator-reduced light, PD = predator-reduced dark, DL = diluted light, VL = virus-reduced light [file 40168_2025_2182_MOESM6_ESM.tif]

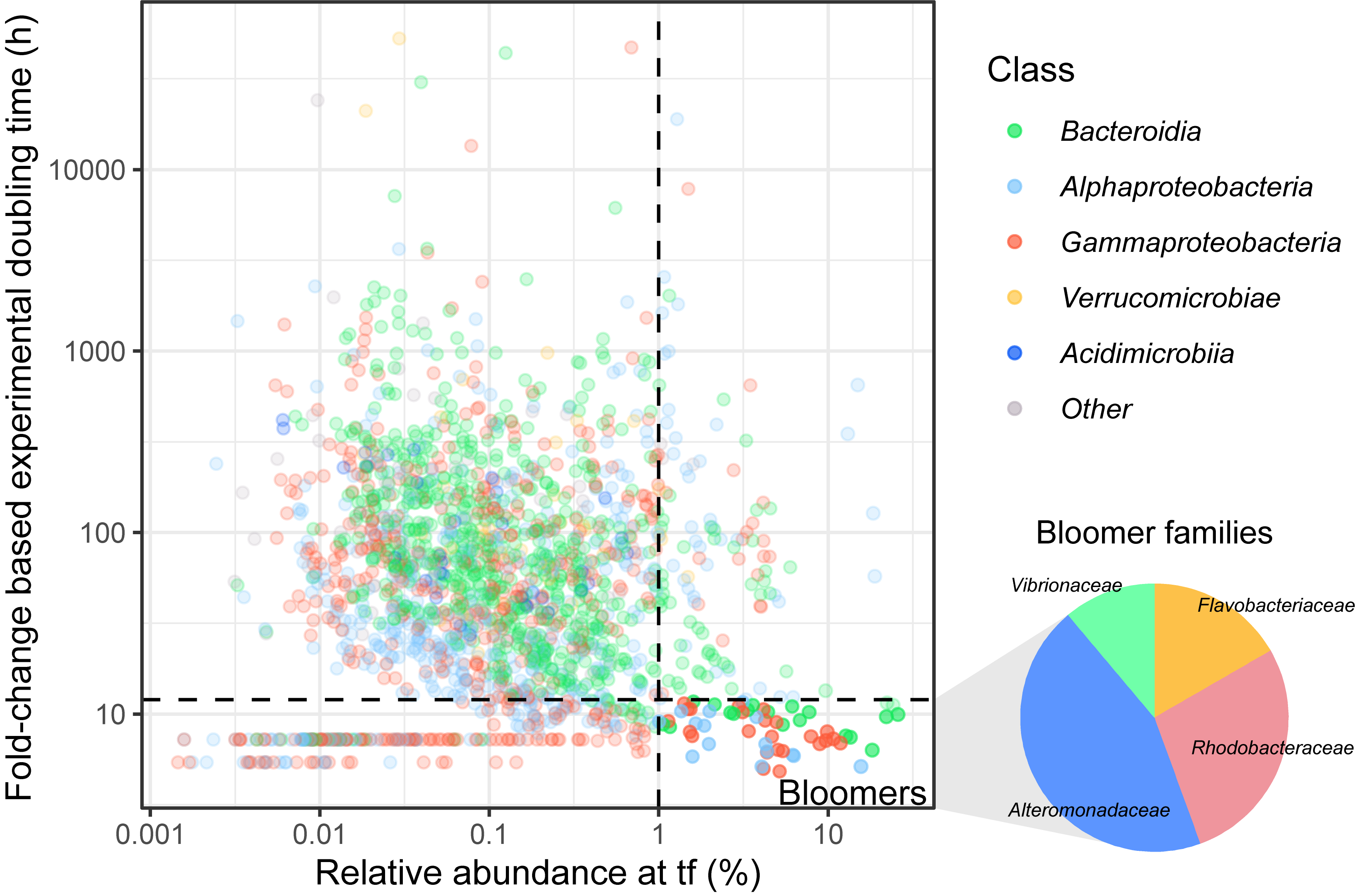

Supplement: Supplementary file 8 — Additional file 7: Figure S7. Relationship between doubling times and relative abundance of each genome at the final time of the experiments. Those which reached fold-change based experimental doubling times of > 12 h and final relative abundances of > 1% were classified as bloomers. A pie chart outlines the family distribution of these bloomers [file 40168_2025_2182_MOESM7_ESM.tif]

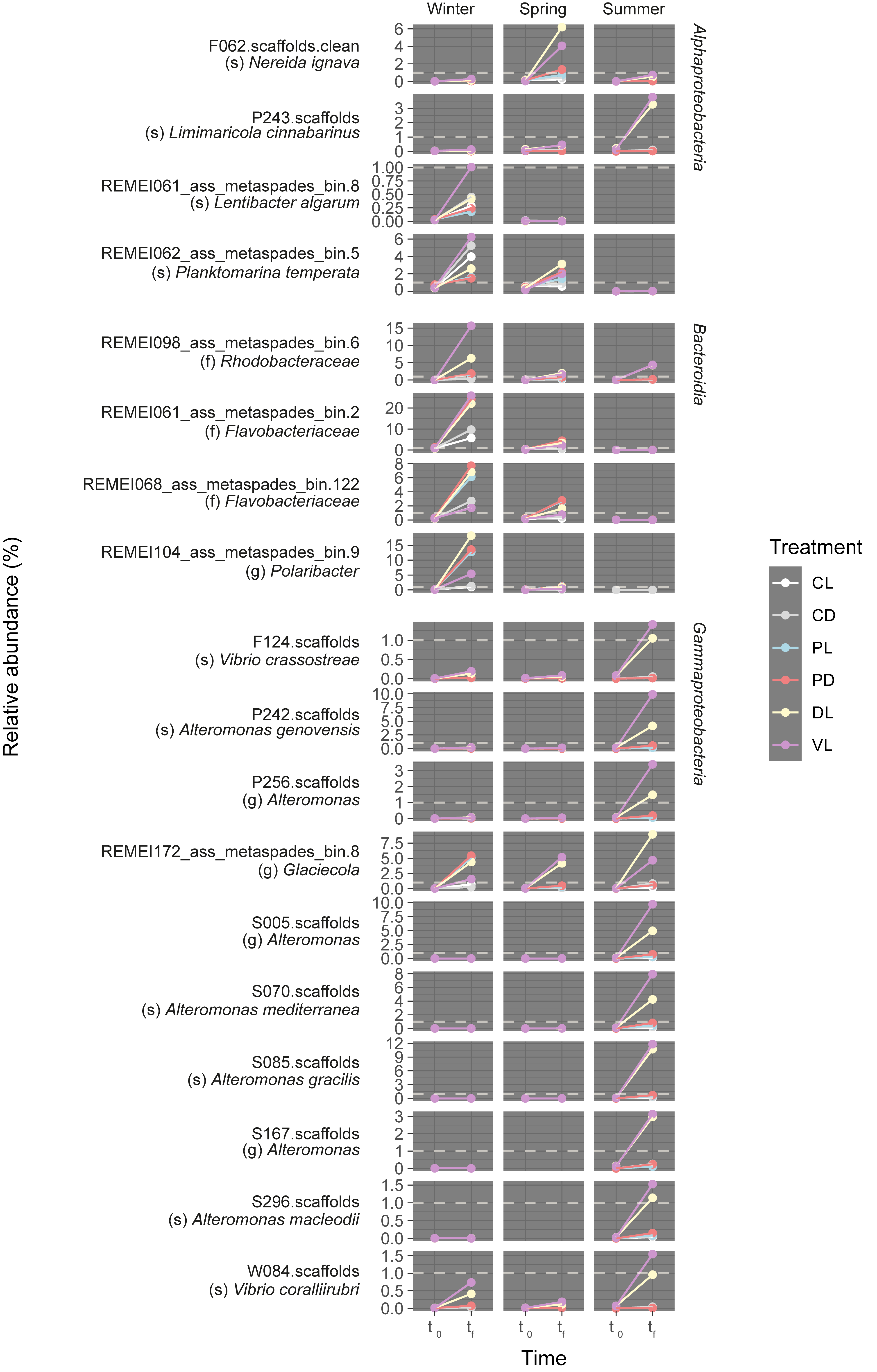

Supplement: Supplementary file 9 — Additional file 8: Figure S8. Abundances of bloomers across treatments and seasons. Relative abundances in the initial (t0) and final times (tf; 36 h in winter and summer, 48 h in spring) of the genomes designated as bloomers in this study in each season and treatment. The Genome IDs and their GTDB taxonomic classification up to the highest level without placeholders, are also indicated. f = family; g = genus; s = species; CL = control light; CD = control dark; PL = predator-reduced light; PD = predator-reduced dark; DL = diluted light; VL = virus-reduced light [file 40168_2025_2182_MOESM8_ESM.tif]

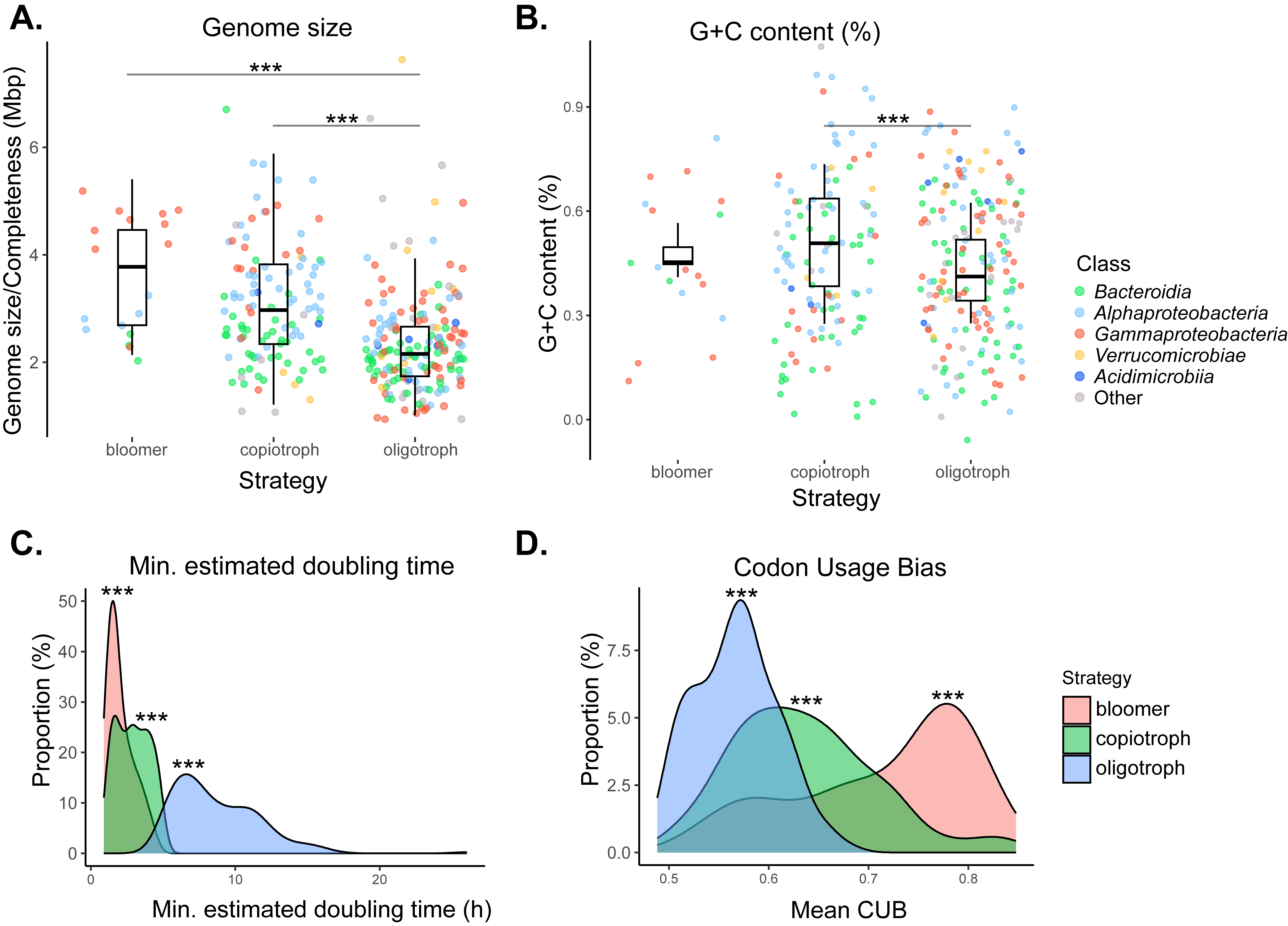

Supplement: Supplementary file 10 — Additional file 9: Figure S9. Comparison of the genomic properties of bloomers, copiotrophs and oligotrophs.A. Genome sizes across strategies presented as boxplots. B. G + C content (%) across strategies. C. Density plots of estimated minimum doubling times (EMDTs) across strategies. D. Density plots of codon usage bias (CUB) across strategies. *** = Pairwise Wilcoxon Rank Sum test p < 0.001 [file 40168_2025_2182_MOESM9_ESM.tif]
